# In Search of the *jüdische Typus*: A Proposed Benchmark to Test the Genetic Basis of Jewishness Challenges Notions of “Jewish Biomarkers”

**DOI:** 10.3389/fgene.2016.00141

**Published:** 2016-08-05

**Authors:** Eran Elhaik

**Affiliations:** Department of Animal and Plant Sciences, University of SheffieldSheffield, UK

**Keywords:** Jewish *Urtypus*, Jewishness, ancestry, Jews, genetic genealogy

## Abstract

The debate as to whether Jewishness is a biological trait inherent from an “authentic” “Jewish type” (*jüdische Typus*) ancestor or a system of beliefs has been raging for over two centuries. While the accumulated biological and anthropological evidence support the latter argument, recent genetic findings, bolstered by the direct-to-consumer genetic industry, purport to identify Jews or quantify one’s Jewishness from genomic data. To test the merit of claims that Jews and non-Jews are genetically distinguishable, we propose a benchmark where genomic data of Jews and non-Jews are hybridized over two generations and the observed and predicted Jewishness of the terminal offspring according to either the Orthodox religious law (Halacha) or the Israeli Law of Return are compared. Members of academia, the public, and 23andMe were invited to use the benchmark to test claims that Jews are genetically distinct from non-Jews. Here, we report the findings from these trials. We also compare the genomic similarity of ∼300 individuals from nearly thirty Afro-Eurasian Jewish communities to a simulated *jüdische Typus* population. The results are discussed in light of modern trends in the genetics of Jews and related fields and provide a tentative answer to the ageless question “who is a Jew?”

## Introduction

While seemingly restricted to a minute fraction of the population, questions regarding Jewish origin and their biological and societal classification have had a profound impact on the arenas of science and politics since their inception (Wexler, 1993; Gissis, 2008; Sand, 2009; Elhaik, 2013; Falk, 2014; McGonigle, 2015; Das et al., 2016) and have lately driven the dramatic rise of the direct-to-consumer (DTC) genetic industry (Slepkov, 2014). First raised by 19th century Jewish scholars like Moses Hess who stated that “Jews are first of all a race” (Hess, 1862), the issue of biological Jewishness immediately became a bone of contention dividing thinkers, scientists, judges, religious leaders, and politicians to this day. While the basic question is ostensibly plain — is Jewishness a heritable trait, like hair color, or a system of beliefs, which anyone can potentially acquire — its relevance to people’s lives and inextricability from contentious topics raised concerns that research in this field is highly subjective (e.g., Azoulay, 2003; Kirsh, 2003). To understand why, it is necessary to consider the racial origins of this question dating back to the 19th century.

Though *scientific racism* can be traced as far back as the Greco-Roman Era (Isaac, 2004), ironically it made its breakthrough into the collective consciousness only during the Age of Enlightenment when questions regarding social equality, economic status, culture, religion, and political inclinations were examined through a biological lens (Efron, 1994). Originally proposed as a biologically meaningless term representing a within-species sub-group, “race” came to denote a biologically distinct social group (Falk, 2014) necessitating the establishment of classification methods that fit people into racial groups. The question of a Jewish race is therefore nearly as old as the question of human races, although it was never entirely clear to which race or races Jews belonged, if at all (Patai and Patai, 1975; Efron, 1994). Establishing an infallible classification required comparing the anthropomorphic traits of Jews with those of other populations in search of their progenitors. Alongside the methodological difficulties underlying such an analysis (e.g., choosing objective traits that remained fixed over time), an unforeseen ideological difficulty soon emerged. Heinrich Graetz’s historical studies of the 1850s proclaimed the Jewish communities as a cohesive “Jewish people” who descended from the Biblical Hebrews. These studies were enthusiastically received by both rabbinical authorities and Zionist leaders (Sand, 2009) and set the course in search for the archaic Jewish type, a *jüdische Typus.* The pursuit of this Jewish archetype led astray many scientists who made bizarre claims about racial differences based on meager differences in anthropomorphic traits, like skull index, while ignoring the vast similarities between Jews and their neighboring populations (Efron, 1994). Physical anthropology not only failed to identify the *jüdische Typus* but also provided unsatisfactory explanations for the vast heterogeneity between and within Jewish communities that seemed to be the norm rather than the exception. Disconcertingly enough, only Yemenite and Palestinian Jews exhibited some evidence of a Mediterranean origin (Patai and Patai, 1975).

Following the rise of hematology and genetics in the mid-20th, the pursuit for the *jüdische Typus* was intensively resumed with a major conceptual adjustment. Abandoning any desire to identify a live *jüdische Typus* specimen, the new search paradigm consisted of identifying common features among contemporary Jews and deriving the characteristics of the *jüdische Typus* from *them* under the presumption that they remained constant through time. However, like their anthropometric predecessors all the “Jewish biomarkers” – blood groups, serum and red cell proteins, “Jewish genes” related to diseases of deficiencies, microsatellites, and even Y chromosomal mutations hailed as “Cohen” and “Levite” “modal haplotypes,” associated with the Biblical priestly class – have failed to embody the yearned-for hallmarks of Jewishness. The reason for the failure was that none of these biomarkers were neither unique nor shared among most Jews – criteria felt necessary to establish their validity (Patai and Patai, 1975; Ritte et al., 1993; Zoossmann-Diskin, 2006; Klyosov, 2009; Tofanelli et al., 2009; Lipphardt, 2010; Zoossmann-Diskin, 2010; Falk, 2014). Even more recent claims that the genetic data support “a common genetic origin, which is consistent with an historical formulation of the Jewish people as descending from ancient Hebrew and Israelite residents of the Levant” (Behar et al., 2010) or even a “Middle Eastern affinity for Indian Jewish populations” (Chaubey et al., 2016) remain unsubstantiated by identifiable mutations and are rather the products of *ad hoc* choices of data partitioning techniques followed by a creative interpretation of the results.

Nonetheless, since these questions have never been settled, the possibility that there exists a “Jewish gene” or certain mutations inherited directly from the *jüdische Typus* receives as much attention nowadays as it received centuries ago (e.g., Tenenbaum and Davidman, 2007; Efron, 2013). Though widely refuted (e.g., Tofanelli et al., 2009), Skorecki et al.’s (1997) study alluding to the existence of mutations on the Y chromosome inherited from Biblical priests was one of the catalysts that simulated the formation of DTC genetic companies (FamilyTreeDNA, 2015). Similar claims that Ashkenazic Jews have originated from “only four women carrying distinct mtDNAs that are virtually absent in other populations” that are “likely from a Hebrew/Levantine mtDNA pool” (Behar et al., 2006) are routinely used to promote genetic products (e.g., Moore, 2012), although these lineages are not unique to Ashkenazic Jews (Tofanelli et al., 2014; Das et al., 2016), are found only in a mere quarter of Ashkenazic Jews (Das et al., 2016), and did not originate in the Middle East (Costa et al., 2013). Soon, an innovation to the binary concept of Judaism was introduced by DTC companies purporting to deduce “whether or not and to what degree you may have Jewish ancestry” (23andMe, 2015). Stated differently, DTC companies proclaimed to report the genomic fraction shared with the *jüdische Typus* – ultimately represented by a selected group of Ashkenazic Jews. Claims that Jews can be accurately distinguished from non-Jews (e.g., “there is a perfect genetic corollary of Jewish ancestry which, in principle, would permit near perfect genetic inference of Ashkenazi [sic] Jewish ancestry” (Need et al., 2009)) and carry “Jewish heritage” in their DNA [“it is clear that the genomes of individuals with full Ashkenazi Jewish ancestry carry an unambiguous signature of their Jewish heritage” (Need et al., 2009)] are also frequently made.

Supporters of the alternative school have consistently dismissed any racial notion of Jews over the past centuries, citing the ongoing failures to provide a robust test for Jewishness and the rich historical, archeological, and linguistic evidence for Jews’ history of assimilations and mixtures with non-Jewish populations rather than seclusion periods (Patai and Patai, 1975; Wexler, 1993; Efron, 1994; Corcos, 2005; Sand, 2009; Falk, 2014). This position can be summarized as: “A Jew is a Jew because he chose to be a Jew and not because he was forced – because of biology or by some external social force – to define himself as a Jew” (Yehoshua, 2013).

Here, we propose a blind-experiment to evaluate claims about the existence of genomic Jewishness (“Jewish genes”) by hybridizing genomic sequences from self-reporting Jews and non-Jews over two generations, and comparing their Jewishness affiliation with that predicted by an examiner blinded to their affiliation. We report the results of a public “Jewish genome challenge” held in late 2013, where members of academia, the public, and industry were asked to infer the Jewishness affiliation of the terminal offspring from their genomic sequences. We also compare the genomic similarity of ∼300 individuals from nearly thirty Afro-Eurasian Jewish communities with a simulated *jüdische Typus* population to test which Jewish community may be traced to it.

The implications of our findings are wide-ranging. If Jewishness is genetic, i.e., there exist genomic mutations that segregate exclusively among all or most Jews, it would contradict over 3,000 years of historical, archeological, and linguistic findings. These findings portray the emergence of Jews from a heterogeneous assortment of Afro-Eurasian tribes who, for centuries and up to this day, have absorbed an enormous number of proselytes around the world. It would allow developing tests to ascertain cryptic Jews or select for or against carriers of “Jewish mutations.” If Jewishness is not genetic, then many of the findings depicting “Jewish genes” or “priestly haplotypes” reported over the past half a century should be reevaluated. Ongoing efforts to study Jews as a distinct group such as the “Jewish HapMap Project” (Ostrer, 2012) or disease studies where adherents of Judaism are over represented due to their presumed genetic homogeneity should also be treated with caution due to the potential biases of population stratification. Our findings will also have implications to the perennial question “who is a Jew?” that has vexed the state of Israel since its establishment.

## Materials and Methods

### Sample Collection

To generate the offspring of Jewish and non-Jewish individuals, we obtained a combined dataset of 1,287 unrelated individuals genotyped by the HGDP (Conrad et al., 2006) and Behar et al. (2010) from http://www.evolutsioon.ut.ee/MAIT/jew_data/ (last accessed December 19, 2012). The dataset consists of 75 non-Jewish populations and 12 Jewish communities (**Supplementary Table S1**), genotyped over 531,315 autosomal and X chromosomal single nucleotide polymorphisms (SNPs). A linkage disequilibrium (LD)-pruned data set was created by removing one member of any pair of SNPs in strong LD (*r*^2^> 0.4) in windows of 200 SNPs (sliding the window by 25 SNPs at a time) using indep-pairwise in PLINK (Purcell et al., 2007). This yielded a total of 226,836 SNPs.

To estimate the relatedness between Jewish communities and the *jüdische Typus* we obtained genotype data from different Jewish communities from recent publications (Behar et al., 2010, 2013; Lazaridis et al., 2014) (**Supplementary Tables S2** and **S3**). After removing individuals representing single communities, 280 individuals from 26 Jewish communities remained. From these datasets, we analyzed only the ∼100,000 autosomal markers that overlapped with the GenoChip markers (Elhaik et al., 2013).

### Forward Simulation

Thirty males and females, half of whom were self- identified as Jews, were randomly selected from the first dataset and randomly paired. Recombination “hot spots” were uniformly positioned every 10,000 markers. Offspring were created by hybridizing regions within recombination hotspots where each parent contributes a random haplotype. Each pair had four offspring, whose sex was randomly determined. Thirty male and female offspring were randomly paired in the next generation. This procedure was followed for two generations. Individual Jewishness was determined by the Orthodox religious law (Halakha; a Jew is anyone whose mother is a Jew) and the Israeli Law of Return of 1970 (a Jew is anyone with at least one Jewish grandparent).

**FIGURE 1:**
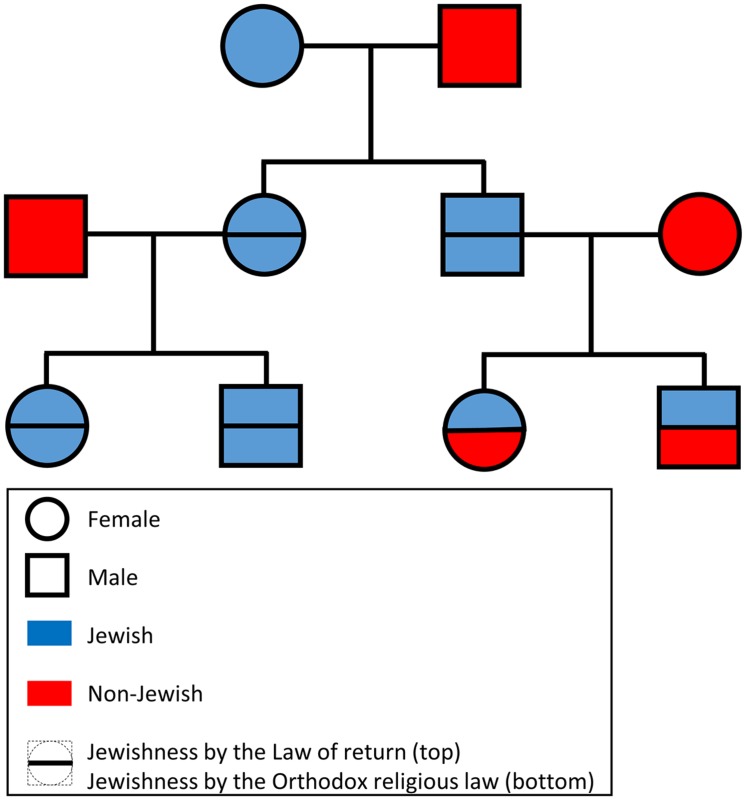
**A schematic diagram of the proposed benchmark.** For brevity only offspring selected to mate in the next generation are shown. The DNA of the terminal offspring was made available to the participants of the “Jewish genome challenge.”

Provided only with the genetic data in PLINK format (Purcell et al., 2007), participants of the “Jewish genome challenge” were asked to predict individual Jewishness according to either law (**Figure 1**). For example, if the male offspring of a non-Jewish African male and a Yemenite Jewish female was hybridized with a non-Jewish Polish female, their offspring would be considered Jewish according to the Law of Return but not according to Orthodox religion law. The participants were asked to provide the answers within 30 days. They were also ensured that their names would not be published in case of a failure and that the correct annotation would be published at the end of the challenge. A webpage was created to inform participants of the benchmark and to make the initial dataset and final results available. The benchmark was advertised over several Facebook groups, Twitter, and popular news websites as a “Jewish genome challenge” (e.g., Hart, 2013; Rosenberg, 2013). Researchers from the field of Jewish genetics were personally invited to participate by email. Finally, 23andMe, a DTC company that reports the proportion of Ashkenazic Jewish ancestry to public participants, was approached.

### Calculating Genetic Distances between Jews and the *jüdische Typus*

**FIGURE 2:**
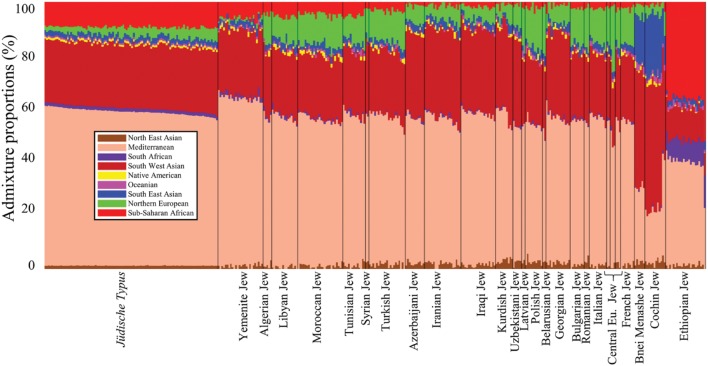
**Admixture proportions of all populations included in this study.** The *x*-axis represents individuals. Each individual is represented by a vertical stacked column of color-coded admixture proportions that reflects genetic contributions from nine putative ancestral populations.

We estimated the genetic similarity between Jewish communities and the *jüdische Typus* by comparing their admixture genomic distance (*d*) defined as the minimal Euclidean distance between the admixture proportions of an individual to those of all individuals of a certain population (Das et al., 2016). To obtain the admixture components, we applied a *supervised* admixture analysis to the dataset of Jewish communities (**Supplementary Tables S2** and **S3**) as described in Elhaik et al. (2014). In such analysis, the nine admixture components of each individual are computed with respect to nine putative ancestral populations whose genomes correspond to those of native populations from different parts of the world. Having a panel of putative ancestral populations allows deriving the same admixture components for individuals of various cohorts. The admixture components of the simulated Israelite populations (i.e., the *jüdische Typus*) were obtained from Das et al. (2016). These admixture components were derived from the same nine admixture distributions reported by Elhaik et al. (2014) and used in this study (**Figure 2**). By projecting the distribution of admixture components along geographical coordinates, Das et al. (2016) were able to identify a range of admixture values that overlapped the borders of modern Israel. They then randomly selected these values for each admixture component and normalized them to sum to 1. These components formed the nine admixture components of individuals that represent native Israelites. The authors also showed that the admixture signatures can be accurately predicted to Israel using GPS, a biogeographical application that converts genomic data into geographic coordinates (Elhaik et al., 2014). A graph illustrating the distances (*d*) between Jews and the *jüdische Typus* population was plotted using Matlab’s *Graph* function.

## Results

Given the large number of scientific studies proclaiming that Jews are distinguishable from non-Jews (e.g., Need et al., 2009), we were surprised that only two individuals accepted the challenge. Both individuals have failed to correctly identify even a single Jewish individual. No criticism on the benchmark was received. 23andMe’s representative refused to participate in the challenge.

### Assessing the Distances between Jews and the *jüdische Typus*

The biological Jewishness school assumes that there exist Jewish biomarkers among all or most contemporary Jews that are shared with the *jüdische Typus*. The successful transmission of these biomarkers over time could only be possible through intrafaith marriages, which implies that all or most contemporary Jews are genetically similar to each other and to the *jüdische Typus*. Failing to satisfy these assumptions dwarves the chances to pass the proposed benchmark.

To test whether passing the benchmark is theoretically possible, we next sought to assess the genetic distance (*d*) within Afro-Eurasian Jewish communities and between these communities and the *jüdische Typus.* Communities originated directly from the *jüdische Typus* population are expected to exhibit short genetic distances to it. Due to the peculiar absence of Israelite ancient DNA that could have shed light on the genomic characteristic of ancient Judaeans, we used simulated admixture data of a highly localized Israelite population that can be considered a proxy to the *jüdische Typus* (**Figure 2**). The admixture proportions of ∼300 individuals belonging to nearly 30 Jewish communities were estimated in respect to nine admixture components corresponding to putative ancestral populations (**Figure 2**).

The *jüdische Typus* genome consists of four major admixture components: Mediterranean (57.7 ± 1.2), South West Asian (23.9 ± 0.6), Sub-Saharan African (9.4 ± 0.5), and Northern European (3.2 ± 1.3). These are also the dominant components among North African (55.7 ± 1.6, 23.5 ± 1, 4.9 ± 1, and 10.4 ± 1.5, respectively), Syrian (55 ± 1, 26.7 ± 0.8, 2.3 ± 0.2, and 10.8 ± 2.3), and Turkish Jews (55.3 ± 1.9, 24.5 ± 1.2, 3.2 ± 0.9, and 11.9 ± 1.2), though their size largely varies. West Eurasian and Near Eastern communities exhibit a smaller Sub-Saharan African fraction (52.8 ± 1.9, 27 ± 1.1, 1.8 ± 0.8, and 12 ± 1.7), unlike Yemenite (62.8 ± 1.1, 24.5 ± 1.3, 6.1 ± 1.8, and 1 ± 0.7) and Ethiopian Jews (38.4 ± 3.6, 12 ± 1, 36.9 ± 3.5, and 0 ± 0.1) that have the highest Sub-Saharan African components. Indian Jews also differ in their admixture pattern (25.4 ± 4.3, 44.7 ± 2.9, 1.2 ± 0.8, and 4.1 ± 1.7) most notably due to their high South East Asian component (19 ± 2.6) compared to all the other Jewish communities (2.1 ± 0.8).

**FIGURE 3:**
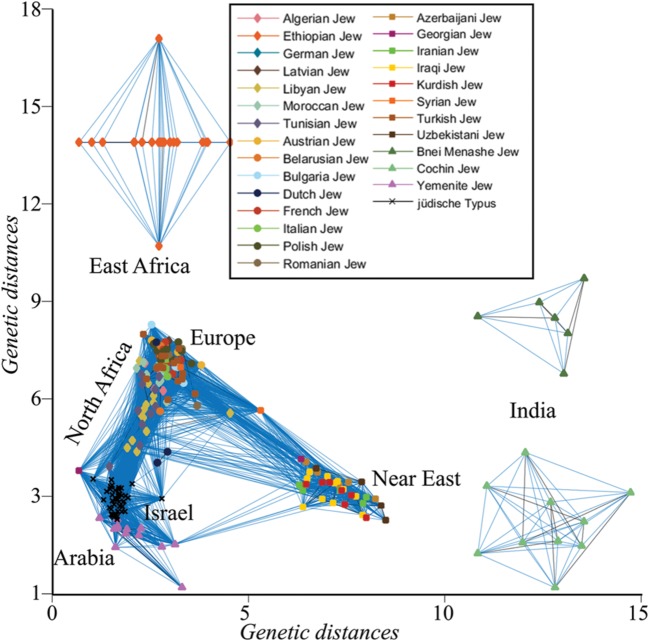
**Undirected graph illustrating the genetic distances (*d*) between Jewish individuals and the *jüdische Typus*, a simulated Israelite population.** For coherency, edges are shown between genetically similar individuals.

The genetic similarity between all individuals was assessed by calculating *d* between all individuals. For brevity, only short distances (*d* < 0.075) were plotted, singling out Indian and Ethiopian Jews that exhibit larger distances to the remaining individuals who cluster along an ‘A’-shaped structure with the ends corresponding to Arabian and Near Eastern Jews. European and Turkish Jews, due to their large number, formed the apex of the ‘A,’ connecting North African with Near Eastern Jews (**Figure 3**). Only a single Yemenite Jew overlapped with a member of the *jüdische Typus* population. Unsurprisingly, the distances of all the Jewish communities from the *jüdische Typus* roughly correspond to their geographical distances from Israel. These results are at odds with the assumptions of the biological Jewishness school.

## Discussion

Biological Jewishness emerged in the late 19th century and quickly became the leading scientific dogma despite its failure to furnish an empirical evidence for over two centuries. Although initially aiming to trace the origin of Jews, the pursuit of biological uniqueness among Jews quickly became a search for a biological link to the *jüdische Typus*, an idealized Jewish type representing the archetype of the ancient Judaeans or Hebrews. This pursuit has never ceased despite the constant failures, with seekers routinely embarking on new scientific technologies, oftentimes driving innovations themselves, claiming each time to unearth the hallmark of biological Jewishness, only to be disappointed again and again (Efron, 1994, 2013; Falk, 2014).

Even today claims such as “the evidence for biological Jewishness has become incontrovertible” (Ostrer, 2012) are frequently made by geneticists and DTC genetic companies though unsupported by the evidence (Lewontin, 2012; Falk, 2014). To evaluate the validity of these claims, we proposed a benchmark to assess whether genomic data can predict Jewishness. Our benchmark consists of genomic data of Jews and non-Jews hybridized over two generations allowing claimants to prove their ability to predict the Jewishness status of the terminal offspring (**Figure 1**). Our benchmark was made publicly available in 2013 as a “Jewish genome challenge” to members of academia, the public, and industry who support the notion of biological Jewishness. The two individuals who attempted to predict the Jewishness status of the terminal offspring have failed.

### Can There Be a Biological Jewishness?

Our findings suggest that claimants of biological Jewishness make assertions that they are unwilling or unable to back in a blinded-experiment, but they do not prove the absence of biological Jewishness. Testing whether such genomic feature or trait exists requires sequencing the genomes of ancient Judaeans and meticulously comparing them with modern day Jewish and non-Jewish individuals, looking for biomarkers that are uniquely shared with the Jewish cohort. However, as our second analysis implies (**Figure 3**), the potential outcomes of such experiment make it unlikely to be performed. In such case, we can ask whether it is reasonable to expect a Jewish biomarker to exist.

Archeological evidence and recent historical thinking suggests that the first Israelites grew out of the sedentarization of local Canaanite populations and nomadic pastoral tribes of unknown origin drifting into Palestine sporadically in the late Bronze Age (Finkelstein and Silberman, 2002; Frendo, 2004). Interestingly, dietary restrictions were the sole difference from their neighboring tribes, as no swine remains were found in their campsites. It is unclear when these tribes have adopted Judaism, which was formulated with strict rules against exogamy much later, but it is reasonable to assume that the traditional bride-exchange, still common among modern day Bedouin tribes (e.g., Bailey, 2009), took place until then. Moreover, it is unlikely that the religion gained wide public acceptance until the days of Ezra and Nehemiah (5th century BC; Patai and Patai, 1975; Patai, 1990; Sand, 2009). Even after this period, the Judaeans remained under the influence of other cultures and faiths and exchanged genes with neighboring and invading populations. As long as Patrilineal Jewishness was the norm, offspring born to either Jewish or non-Jewish mothers were considered Jews through their paternal descent. It was not until around the 2nd century AD that the offspring’s Jewishness was determined by the matrilineal descent (Sigal, 1985). The Judaeans have been proselytizing their neighbors from their early days, but these activities intensified during the early centuries A.D. and encompassed several Old World populations (Patai and Patai, 1975; Sand, 2009). Even after the rise of Christianity (4th century AD) and Islam (7th century AD), the Judaisation of slaves continued for several centuries until banned by the religious authorieties (Sand, 2009). The last notable conversion took place in Khazaria in the mid-8th century AD and included the Khazarian elite and some of the Khazar people (Sand, 2009; Elhaik, 2013). Massive conversions to Judaism were renewed only after the establishment of the state of Israel (DellaPergola, 2005).

The Israeli Law of Return that passed in 1950, but was corrected in 1970, provided Israeli citizenship to anyone who had one Jewish grandparent or a Jewish partner (similarly defined as anyone with one Jewish grandparent). The law was criticized for allowing non-Jews to become citizens, but it allowed Israel to absorb an enormous number of immigrants, primarily from the former Soviet Union and Ethiopia, many of whom converted to Judaism (DellaPergola, 2005; Cook, 2015). The recent drop in the number of immigrants prompted Israel to consider relaxing its migration policy to allow “groups with ties to the Jewish people” to immigrate to Israel assuming that “with proper indoctrination and incentives, non-Jews who are not Arab can be tempted to immigrate and add to the [demographic] balance sheet” (Cook, 2015). The newcomers did not remain socially isolated for too long. During the early 1990s, Israelite Jews had low consanguinity rates (2.3%) and high intracommunity marriages (64%), and both have been declining in favor of intercommunity marriages (Cohen et al., 2004). These rates are so high that the Israeli Minister of Health has recently recommended to scrap the use of “ethnic categories” (e.g., “Iraqi Jews”) and offer genetic tests for all Jewish couples (Even, 2012).

Major demographic shifts also took place in the other side of the ocean where the second largest Jewish population lives. In 1983, Reform Judaism, the leading movement among US Jews, acknowledged the Jewishness of both matrilineal and patrilineal descent, which nearly eliminated the necessity to convert to Judaism. Today, the intermarriage rate in the US is 58% for all Jews and 71% for non-Orthodox Jews (Lugo et al., 2013) with similarly higher rates in Europe (55–75%; DellaPergola, 2003). The smaller Conservative and Orthodox movements maintain loyalty to the matrilineal descent and reject out-marriage (DellaPergola, 2005). Movements into Judaism were almost always followed by counter movements out of Judaism (DellaPergola, 2003).

These demographic shifts allow us to derive six reasonable conclusions:

(1) It is unlikely that an “Israelite gene” ever existed since Iron Age Israelite tribes exchanged genes with their neighboring tribes.
(2) Had there been a paternal “Israelite gene” on the Y chromosome, it would have been lost due to the transition to matrilineal descent.
(3) Had there been a maternal “Israelite gene” on the mitochondrial chromosome, it would have been lost due to the initial period of partilineal descent.
(4) Had there been an autosomal “Israelite gene,” it would have been lost due to the high rates of movements into the religion.
(5) Had there been an autosomal “Israelite gene,” it would not be unique to Jews due to the high rates of movements out of the religion.
(6) Had there been an autosomal “Israelite gene” that survived to modern days, it would have been extremely rare and undetectable by popular search approaches that prioritize findings common to a large fraction of Jews.

In our second analysis, we showed that Jewish communities are genomically distinct from each other (primarily Africa, Arabia, Near Eastern, and European Jews) and from the simulated *jüdische Typus*, as one can expect from their history – in agreement with our conclusions and the literature (e.g., Patai and Patai, 1975; Kaplan, 2003). Interestingly, in a recent ancient DNA analysis of six Natufians and a Levantine Neolithic (Lazaridis et al., 2016), some of the likely Judaean progenitors (Finkelstein and Silberman, 2002; Frendo, 2004), the ancient individuals clustered predominantly with modern-day Palestinians and Bedouins and marginally overlapped with Arabian Jews. Ashkenazic Jews clustered away from these ancient Levantine individuals and adjacent to Neolithic Anatolians and Late Neolithic and Bronze Age Europeans.

### Evaluating the Evidence of Genetic Jewishness

If Jewishness is not biological, it is of interest to critically assess the scientific framework, particularly the assumptions, arguments, methodology, and conclusions used to support the counter argument. Interestingly, a review of genetic studies carried out between 1951 and 1963 concluded that Zionist ideas had a negative impact on the objectivity of genetic research (Kirsh, 2003). To the best of our knowledge, a critical evaluation of recent studies, particularly those employing genome-wide analyses, was never compiled. While a comprehensive review is beyond the scope of this paper, we highlight four problems with contemproary research.

#### (1) Reinforcing Jewish Nationalist Narratives in Diseases Studies

Categorizing pathogenic mutations associated with devastating diseases as “Jewish” or “Ashkenazic” is a powerful means to promote a global Jewish national identity, as it supports the argument that Jews have emerged from a small homogenic cohort, allegedly from a *jüdische Typus*. “Jewish diseases” serve dual roles by enforcing ideas of “racial hygiene” (Sand, 2009) or Eugenics and identifying “cryptic Jews.” For example, mutations in the *BRCA1* and other genes leading to breast cancer were initally dubbed “Ashkenazic mutations” and were intepreted as evidence to a shared Judaean or Palestinian past (Ostrer, 2001). This label was not questioned when these mutations were found in high frequency among Hispanic Americans. Instead, it has been proposed that they descended from Spanish Jewish *conversos*, who were forced to abandon their religion in the 15th century. A similar interpretation was made when Native Americans from Colombia were also found to be carriers. It later became clear that a large number of mutations exist among women who do not fit any local genetic testing criteria and that screening programs should be extended (reveiwed by Mozersky and Gibbon, 2014). Even the relative abundance of rare diseases like Tay-Sachs, Neimann-Pick, and Gaucher has been convincingly explained by historic epidemics of tuberculosis in industrial European cities (O’Brien, 1991) rather than a shared genetic legacy among all Jewish communities.

#### (2) Adhering to Ludicrous Histocial Scenarios

Conversions, assimilations, and later acceptance of individuals of non-Jewish backgrounds or even those with remote Jewish background into Jewish communities allowed Judaism to grow and expand throughout the Old World and sustain the Jewish population in Israel. These demographic processes contrast notions of imagined genomic homogeneity, which does not even exist within Judaean skeletons (Haas, 1970). The arguments in favor of genomic homogeneity are supported by two fictitious chapters in Jewish history (Sand, 2009). The first chapter tells of the “Roman Exile” that followed the destruction of Herod’s temple (70 AD) and introduced a massive Jewish population to Roman lands (e.g., King, 2001). The second chapter tells of a small group of 50,000 Jews who migrated from the Rhineland to Eastern Europe in the 15th century and then rapidly reproduced to 5,000,000 people at the beginning of the 19th century, forming the bulk of Ashkenazic Jews where: “entry into the community was possible through religious conversion, but that was not common. Jewish identity was maintained within these communities up to present day” (Ostrer, 2012). Since such an unnatural growth rate over half a millennium applying only to Eastern European Jews within this specific time period cannot be explained (van Straten and Snel, 2006) it has been termed a “demographic miracle” (Ben-Sasson, 1976; Atzmon et al., 2010; Ostrer, 2012). The assumption of biological Jewishness implied from these chapters forms the rationale for nearly every genetic investigation that focuses on Jews or more commonly Ashkenazic Jews (e.g., Bray et al., 2010; Goes et al., 2015).

Following Huxley (1873) who stated that: “It is, indeed, a conceivable supposition that every species of Rhinoceros and every species of Hyæna, in the long succession of forms between the Miocene and the present species, was separately constructed out of dust, or out of nothing, by supernatural power; but until I receive distinct evidence of the fact, I refuse to run the risk of insulting any sane man by supposing that he seriously holds such a notion,” we too agree that it is a conceivable supposition that the Judaeans were exiled by the Romans and then reproduced via a “demographic miracle,” but until evidence is forthcoming for these two otherwise undocumented theories, such explanations should be dismissed.

Though evidence points to the contrary (e.g., Bray et al., 2010; Listman et al., 2010; Elhaik, 2013) (**Figures 2** and **3**), the alleged genetic homogeneity of Ashkenazic Jews also constitutes the rationale for ancestry testing offered by DTC genetic companies (Durand et al., 2014). In the absence of evidence for biological Jewishness, the accuracy of this design cannot be empirically tested. However, testing a meager number of reference populations, representing a trifling 0.4–0.5% of the estimated 5000–6000 of worldwide populations (Fardon, 2012), as in the case of 23andMe, increases the likelihood that tested individuals will show some relatedness to Ashkenazic Jews.

#### (3) Misinterpreting Patterns in Complex Datasets in Favor of a Narrative

Principal component (PC) and structure-like analyses are the hammer and chisel in large scale analyses, at least partially because they can produce a multitude of resuls in support of various conclusions (McVean, 2009; Parsons et al., 2009; Weiss and Long, 2009; Weiss, 2015). For example, Behar et al. (2010) were able to generate different PCA plots that differ in the overlap of the tested populations by changing the inclusion criteria of the studied populations and treated all the results as equally valid. In a separate analysis, PCA distinguishing between Ashkenazic Jews and “Caucasian” individuals was enthusiastically interpreted in favor of their genetic uniqueness (Need et al., 2009). However, a similar trend would be obtained for any “non-Caucasian” population, and since the DNA of Ashkenazic Jews was shown to have originated in “ancient Ashkenaz” in northeastern Turkey (Das et al., 2016) these results are not surprising.

**FIGURE 4:**
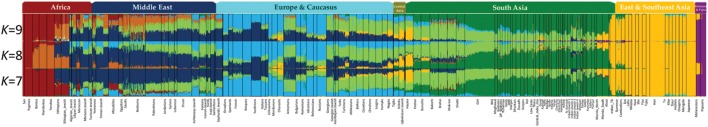
**Admixture analysis of worldwide populations.** Admixture analysis was performed for *K* = 7–9. The *x*-axis represents individuals from populations sorted according to their reported ancestries. Each individual is represented by a vertical stacked column of color-coded admixture proportions that reflects genetic contributions from putative ancestral populations. Indian Jews are underlined. The authors considered the dark blue component visible *K* = 7 as a representative of a “Middle East Ancestry.” Modified from Chaubey et al. (2016).

By contrast to a *supervised* admixture analyses (e.g., **Figure 3**), *unsupervised* admixture consists of making any number of partitions in the allele frequencies of various populations (e.g., *K* = 3 or *K* = 9) summarizing the results so that the frequency *Q* of all partitions or components sums to 1 (e.g., in *K* = 3, 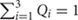), and selecting the most convenient results. For example, Behar et al. (2010) proposed that certain admixture components in *K* = 8, which exist among Israelite populations, are indicative of a Middle Eastern origin for European Jews. However, none of these components was unique to Israelite or Middle Eastern populations. A similar argument was recently made in favor of a Middle Eastern origin for Indian Jews (Chaubey et al., 2016). Yet the chosen “Middle Eastern” admixture component was also found among Africans, Europeans, and South Asians (**Figure 4**, *K* = 7, blue). Remarkably, the only admixture component (**Figure 4**, *K* = 9, brown) that was highly localized to Middle Eastern populations and absent from Indian Jews was ignored.

#### (4) Clinging to Outliers while Ignoring the Overall Trend

There is much similarity between the claims about Jewish group membership made in the 19th century (Efron, 1994) and those made later on. In all cases, authors cling to very small differences in a small number of genomic markers. For example, Spurdle and Jenkins (1996) claimed that over 50% of the Lemba, a southern African Bantu-speaking population claiming Jewish ancestry, are of Semitic origin based on an analysis of four Y chromosomal loci. Excitingly, one of the marker (*p12F2/TaqI*) was found to be absent in Africans, though it existed among various Jewish communities (that comprised 60% of the reference samples) with Yemenite Jews showing the highest frequency. The authors proposed that this marker is “specific to Caucasoids” and is an evidence of a “Semitic origin” for the Lemba people. Similar interpretations were made for all other markers, and although an African origin was oftentimes mentioned as a viable alternative, the “consensus” of the results was a Semitic origin, assumed to be the origin of all Jewish and Middle Eastern communities (together representing 70% of the reference samples). The fallacy here is obvious. Since Jewish communities are highly similar to their neighboring non-Jewish communities and altogether encompass a vast genetic heterogeneity, it is feasable to show that anyone is genetically “close” to at least one Jewish community and argue in favor of the “Semitic origin” of the tested individual.

This reasoning also characterizes the decade old pursuit of “Cohen” and “Levite,” markers. Although dispelled on numerous occasions (Zoossmann-Diskin, 2006; Klyosov, 2009; Tofanelli et al., 2009), the pursuit for priestly biomarkers continued relentlessly to this day ever since Skorecki et al. (1997) embarked on this hare. Hammer et al. (2009) even set their hopes on the idea that these biomarkers might aid in the “identification of lost tribes claiming ancient Hebrew ancestry,” although they exist in almost every non-Jewish population surveyed, none of which have ever claimed a Hebrew priestly descent nor considered themselves lost tribes longing to be found.

There are a few more reasons to doubt that the end results of this pursuit would be different. First, “Cohen” and “Levite” are the two most common surnames among Jews in Israel (1.93 and 1.12%, respectively; Margalit, 2014) and elsewhere (e.g., Kosmin and Waterman, 1985), all of whom cannot possibly be affiliated with the small number of priests serving in the Second Jewish Temple. Second, these surnames are distributed unevenly among Jewish communities. In many Jewish communities, the surname Cohen does not appear at all, while in others almost all members of the congregation are called “Cohen” without a single “Levy,” indicating collective convergence (Sand, 2009). Third, the semantic similarity between Cohen (Kahan) and Khagan, a royalty title used by Turkish people, has been recently postulated to indicate a pagan-Shamanistic background (Das et al., 2016). Some Jewish individuals with “Kohen” and “Kagan” surnames were shown to exhibit genetic similarity (Brook, 2014). Finally, without demonstrating a genomic similarity to priestly and Judaean ancient DNA, interpretations in favor of a these origin are speculative, at best.

### Motives for Genetic Research

While the financial drive behind DTC genetic companies is obvious, several authors raised concerns that much of the subjectivity of genetic research in this field is driven by political motives (e.g., Efron, 1994; Azoulay, 2003; Kirsh, 2003; Kahn, 2005). Efron (1994) cautioned that “unfortunately, much like its racial science predecessor, the recently young field of popualtion genetics is also laden with prejudice and political motivation.” Ostrer (2012) remarked that “The stakes in genetic analysis are high. It is more than an issue of who belongs in the family and can partake in Jewish life and Israeli citizenship. It touches on the heart of Zionist claims for a Jewish homeland in Israel. One can imagine future disputes about exactly how large the shared Middle Eastern ancestry of Jewish groups has to be to justify Zionist claims.” Regretfully, Ostrer’s vision is discriminatory against Jews representing 75% of the Israelite population, though they share a smaller Mediterranean or Middle Eastern ancestries of ∼50% (Carmi et al., 2014) (**Figure 2**) compared to native Israelites (56–59%; Das et al., 2016).

There is evidence to indicate that proselytization or even religious affinity, which translates into political support (Slepkov, 2014), is another strong motive. Bennett Greenspan, president and CEO of Family Tree DNA stated that: “many people who learn of Semitic ancestry through DNA often end up converting to Judaism” (Dolgin, 2011). An extreme example of religious conversion is the case of Csanad Szegedi, a former leader of Hungary’s extreme right-wing political party known for his anti-Semitic rhetoric, who converted to Judaism upon learning of his Jewish heritage via a DNA test (Slepkov, 2014). Defining biological Jewishness as a “genetic similarity” between an individual and any self-identified Jew is a recipe for large-scale proselytization of middle-class Anglophones due to the rapidly rising heterogeneity of the “Jewish gene pool” (**Figure 2**) that allegedly traces back to the *jüdische Typus*.

## Conclusion

The humble beginning of the paternally inherited agro-pastoral faith whose essence was largely conceived by Judahite priests during the dawn of the 5th century BC stands in sharp contrast to a depiction of a biological trait passed unaltered from generation to generation withstanding the tests of time, conversions, diseases, and extinctions until finally depicted as a genetic legacy that can discriminate non-Jews from the descendants of the legendary *jüdische Typus*.

Here, we propose the first benchmark to test claims that Jews are genomically distinct from non-Jews. Most members of academia, the public, and industry invited to prove those claims did not partake the benchmark, and those who attempted to identify Jews from genomic data failed. Our study was limited both by the simulated recombination process and the relatively small number of autosomal markers, and although these markers were used to support claims in favor of biological Jewishness, we cannot dismiss the existence of a hypothetical “Jewish marker” elsewhere in the genome. Moreover, our benchmark was designed to infer only a binary notion of Jewishness, corresponding to the law of Return and the Halacha, not the innovative depiction of discrete Jewishness, though such claims also derive from the binary notion of Jewishness.

The growing field of omics offers vast opportunities to search for such biomarkers throughout the genome, exome, methylome, cellome, chromatinome, transcriptome, alleome, proteome, lipidome, interactome, spliceome, ORFeome, phosphoproteome, metabolome, mechanome, epigenome, histome, and phenome. Advances in the antibodyome and auto antibodyome as well as connectome, glycome, and kinome should also be considered. Special attention should also be paid to the physiome and neurome. It is not inconceivable that the microbiome, virome, bacteriome, or overall mycobiome, might be surveyed scrupulously to detect telltale hallmarks of “Jewishness.” However, until robust and reproducible evidence is forthcoming, in which a benchmark of the kind proposed here is employed, the alternative hypothesis must be upheld: Jewishness lies in the socialome.

## Author Contributions

The author confirms being the sole contributor of this work and approved it for publication.

## Conflict of Interest Statement

EE consults the DNA Diagnostic Centre (DDC).

## References

[B1] 23andMe (2015). *Can 23andMe Identify Jewish ancestry*? Available at: https://eu.customercare.23andme.com/hc/en-us/articles/204444434-Can-23andMe-identify-Jewish-ancestry (accessed March 23, 2016).

[B2] Atzmon G. Hao L. Pe’er I. Velez C. Pearlman A. Palamara P. F. (2010). Abraham’s children in the genome era: major Jewish diaspora populations comprise distinct genetic clusters with shared Middle Eastern Ancestry. *Am. J. Hum. Genet.* 86 850–859. 10.1016/j.ajhg.2010.04.015 20560205 PMC3032072

[B3] Azoulay K. G. (2003). Not an innocent pursuit: the politics of a ‘Jewish’ genetic signature. *Dev. World Bioeth.* 3 119–126. 10.1046/j.1471-8731.2003.00067.x 14768643

[B4] Bailey C. (2009). *Bedouin Law from Sinai and the Negev: Justice without Government.* New Haven, CT: Yale University Press.

[B5] Behar D. M. Metspalu E. Kivisild T. Achilli A. Hadid Y. Tzur S. (2006). The matrilineal ancestry of Ashkenazi Jewry: portrait of a recent founder event. *Am. J. Hum. Genet.* 78 487–497. 10.1086/500307 16404693 PMC1380291

[B6] Behar D. M. Metspalu M. Baran Y. Kopelman N. M. Yunusbayev B. Gladstein A. (2013). No evidence from genome-wide data of a Khazar origin for the Ashkenazi Jews. *Hum. Biol.* 85 859–900. 10.3378/027.085.0604 25079123

[B7] Behar D. M. Yunusbayev B. Metspalu M. Metspalu E. Rosset S. Parik J. (2010). The genome-wide structure of the Jewish people. *Nature* 466 238–242. 10.1038/nature09103 20531471

[B8] Ben-Sasson H. H. (1976). *A History of the Jewish People.* Cambridge, MA: Harvard University Press.

[B9] Bray S. M. Mulle J. G. Dodd A. F. Pulver A. E. Wooding S. Warren S. T. (2010). Signatures of founder effects, admixture, and selection in the Ashkenazi Jewish population. *Proc. Natl. Acad. Sci. U.S.A.* 107 16222–16227. 10.1073/pnas.1004381107 20798349 PMC2941333

[B10] Brook K. A. (2014). The genetics of crimean karaites. *Karadeniz Araştırmaları (Journal of the Black Sea Studies).* 42 69–84.

[B11] Carmi S. Hui K. Y. Kochav E. Liu X. Xue J. Grady F. (2014). Sequencing an Ashkenazi reference panel supports population-targeted personal genomics and illuminates Jewish and European origins. *Nat. Commun.* 5:4835 10.1038/ncomms5835 PMC4164776 25203624

[B12] Chaubey G. Singh M. Rai N. Kariappa M. Singh K. Singh A. (2016). Genetic affinities of the Jewish populations of India. *Sci. Rep.* 6 1–9. 10.1038/srep19166 PMC4725824 26759184

[B13] Cohen T. Vardi-Saliternik R. Friedlander Y. (2004). Consanguinity, intracommunity and intercommunity marriages in a population sample of Israeli Jews. *Ann. Hum. Biol.* 31 38–48. 10.1080/0301446032000159255 14742164

[B14] Conrad D. F. Jakobsson M. Coop G. Wen X. Wall J. D. Rosenberg N. A. (2006). A worldwide survey of haplotype variation and linkage disequilibrium in the human genome. *Nat. Genet.* 38 1251–1260. 10.1038/ng1911 17057719

[B15] Cook J. (2015). *Israel Hopes ‘Lost Tribes’ can Boost Jewish Numbers. Global Research.* Available at: http://www.globalresearch.ca/israel-hopes-lost-tribes-can-boost-jewish-numbers/5474174 (accessed March 21, 2016).

[B16] Corcos A. F. (2005). *The Myth of the Jewish Race: A Biologist’s Point of View.* Bethlehem, PA: Lehigh University Press.

[B17] Costa M. D. Pereira J. B. Pala M. Fernandes V. Olivieri A. Achilli A. (2013). A substantial prehistoric European ancestry amongst Ashkenazi maternal lineages. *Nat. Commun.* 4:2543 10.1038/ncomms3543 PMC3806353 24104924

[B18] Das R. Wexler P. Pirooznia M. Elhaik E. (2016). Localizing Ashkenazic Jews to primeval villages in the ancient Iranian lands of Ashkenaz. *Genome Biol. Evol.* 8 1132–1149. 10.1093/gbe/evw046 26941229 PMC4860683

[B19] DellaPergola S. (2003). *Jewish Out-Marriage: A Global Perspective.* Available at: http://archive.jpr.org.uk/object-bjpa222 (accessed March 21, 2016).

[B20] DellaPergola S. (2005). *The Jewish People Policy Planning Institute Annual Assessment 2005: Facing a Rapidly Changing World.* Jerusalem: Jewish People Policy Planning Institute.

[B21] Dolgin E. (2011). *Family Roots. Forward.* Available at: http://forward.com/articles/134758/family-roots/ (accessed March 21, 2016).

[B22] Durand E. Y. Do C. B. Mountain J. L. Macpherson J. M. (2014). Ancestry composition: a novel, efficient pipeline for ancestry deconvolution. *biorxiv* 010512 10.1101/010512

[B23] Efron J. M. (1994). *Defenders of the Race.* New Haven, CT: Yale University Press.

[B24] Efron J. M. (2013). Jewish genetic origins in the context of past historical and anthropological inquiries. *Hum. Biol.* 85 901–918. 10.3378/027.085.0602 25079124

[B25] Elhaik E. (2013). The missing link of Jewish European ancestry: contrasting the Rhineland and the Khazarian hypotheses. *Genome Biol. Evol.* 5 61–74. 10.1093/gbe/evs119 23241444 PMC3595026

[B26] Elhaik E. Greenspan E. Staats S. Krahn T. Tyler-Smith C. Xue Y. (2013). The GenoChip: a new tool for genetic anthropology. *Genome Biol. Evol.* 5 1021–1031. 10.1093/gbe/evt066 23666864 PMC3673633

[B27] Elhaik E. Tatarinova T. Chebotarev D. Piras I. S. Maria Calò C. De Montis A. (2014). Geographic population structure analysis of worldwide human populations infers their biogeographical origins. *Nat. Commun.* 5:3513 10.1038/ncomms4513 PMC4007635 24781250

[B28] Even D. (2012). *Health Ministry: Jews shouldn’t be Categorized by Ethnicity in Genetic Tests.* Haaretz Available at: http://www.haaretz.com/israel-news/health-ministry-jews-shouldn-t-be-categorized-by-ethnicity-in-genetic-tests-1.449997 (accessed March 21, 2016).

[B29] Falk R. (2014). Genetic markers cannot determine Jewish descent. *Front. Genet.* 5:462 10.3389/fgene.2014.00462 PMC4301023 25653666

[B30] FamilyTreeDNA (2015). *Discover your Jewish Ancestry.* Available at: https://www.familytreedna.com/landing/jewish-ancestry.aspx (accessed March 21, 2016).

[B31] Fardon R. (2012). *The Sage Handbook of Social Anthropology.* Thousand Oaks, CA: SAGE Publications 10.4135/9781446201077

[B32] Finkelstein I. Silberman N. A. (2002). *The Bible Unearthed : Archaeology’s New Vision of Ancient Israel and the Origin of its Sacred Texts.* New York, NY: Simon and Schuster.

[B33] Frendo A. J. (2004). “Back to Basics: a holistic approach to the problem of the emergence of ancient Israel,” in *Search of Pre-Exilic Israel*, ed. Day J. (New York, NY: T&T Clark International), 41–64. 10.1097/00152193-200410000-00004

[B34] Gissis S. B. (2008). When is ‘race’ a race? 1946-2003. *Stud. Hist. Philos. Biol. Biomed. Sci.* 39 437–450. 10.1016/j.shpsc.2008.09.006 19026975

[B35] Goes F. S. McGrath J. Avramopoulos D. Wolyniec P. Pirooznia M. Ruczinski I. (2015). Genome-wide association study of schizophrenia in Ashkenazi Jews. *Am. J. Med. Genet. B Neuropsychiatr. Genet.* 168 649–659. 10.1002/ajmg.b.32349 26198764

[B36] Haas N. (1970). Anthropological observations on the skeletal remains from Giv’at ha-Mivtar. *Israel Explor. J.* 20 38–59.

[B37] Hammer M. F. Behar D. M. Karafet T. M. Mendez F. L. Hallmark B. Erez T. (2009). Extended Y chromosome haplotypes resolve multiple and unique lineages of the Jewish priesthood. *Hum. Genet.* 126 707–717. 10.1007/s00439-009-0727-5 19669163 PMC2771134

[B38] Hart A. (2013). *Website Proposes to Hold the Jewish Genome Challenge. examiner.com.* Available at: http://www.examiner.com/article/website-proposes-to-hold-the-jewish-genome-challenge (accessed March 21, 2016).

[B39] Hess M. (1862). *Rome and Jerusalem: The Last National Question.* Leipzig: Eduard Mengler.

[B40] Huxley T. H. (ed.) (1873). “Palaeontology and the doctrine of evolution (The anniversary address to the geological society, 1870),” in *Critiques and Addresses* (London: Macmillan), 181–217.

[B41] Isaac B. H. (2004). *The Invention of Racism in Classical Antiquity.* Princeton, NY: Princeton University Press.

[B42] Kahn S. M. (2005). The multiple meanings of Jewish genes. *Cult. Med. Psychiatry* 29 179–192. 10.1007/s11013-005-7424-5 16249949

[B43] Kaplan S. (2003). If there are no races, how can Jews be a “race?” *J. Mod. Jew. Stud.* 2 79–96. 10.1080/14725880305901

[B44] King R. D. (2001). The paradox of creativity in diaspora: the Yiddish language and Jewish identity. *Stud. Ling. Sci.* 31 213–229.

[B45] Kirsh N. (2003). Population genetics in Israel in the 1950s. The unconscious internalization of ideology. *Isis* 94 631–655. 10.1086/386385 15077535

[B46] Klyosov A. A. (2009). A comment on the paper: extended Y chromosome haplotypes resolve multiple and unique lineages of the Jewish Priesthood by M. F. Hammer, D. M. Behar, T. M. Karafet, F. L. Mendez, B. Hallmark, T. Erez, L. A. Zhivotovsky, S. Rosset, K. Skorecki. *Hum. Genet.* 126 719–724. 10.1007/s00439-009-0739-1 19669163 PMC2771134

[B47] Kosmin B. Waterman S. (1985). *The Use and Misuse of Distinctive Jewish Names in Research on Jewish Populations.* Jerusalem: Hebrew University of Jerusalem.

[B48] Lazaridis I. Nadel D. Rollefson G. Merrett D. C. Rohland N. Mallick S. (2016). The genetic structure of the world’s first farmers. *bioRxiv* 10.1101/059311

[B49] Lazaridis I. Patterson N. Mittnik A. Renaud G. Mallick S. Kirsanow K. (2014). Ancient human genomes suggest three ancestral populations for present-day Europeans. *Nature* 513 409–413. 10.1038/nature13673 25230663 PMC4170574

[B50] Lewontin R. C. (2012). *Is There a Jewish gene?* New York, NY: The New York Review of books Available at: http://www.nybooks.com/articles/2012/12/06/is-there-a-jewish-gene/ (accessed March 21, 2016).

[B51] Lipphardt V. (2010). The Jewish community of Rome: an isolated population? Sampling procedures and bio-historical narratives in genetic analysis in the 1950s. *BioSocieties* 5 306–329. 10.1057/biosoc.2010.16

[B52] Listman J. B Hasin D. Kranzler H. R. Malison R. T. Mutirangura A. Sughondhabirom A. (2010). Identification of population substructure among Jews using STR markers and dependence on reference populations included. *BMC Genet.* 11 48–63. 10.1186/1471-2156-11-48 20546593 PMC2896335

[B53] Lugo L. Cooperman A. Smith G. A. Hackett C. Funk C. Connor N. S. P. (2013). *A Portrait of Jewish Americans: Findings from a Pew Research Center Survey of US Jews Pew Research Institute.* Available at: http://www.pewforum.org/2013/10/01/jewish-american-beliefs-attitudes-culture-survey/ (accessed March 21, 2016).

[B54] Margalit M. (2014). *What Are Most Common Surnames in Israel?* Available at: http://www.ynetnews.com/articles/0,7340,L-4476581,00.html (accessed April 18, 2016).

[B55] McGonigle I. V. (2015). ‘Jewish genetics’ and the ‘nature’ of Israeli citizenship. *Transversal.* 13 90–102. 10.1515/tra-2015-0010

[B56] McVean G. (2009). A genealogical interpretation of principal components analysis. *PLoS Genet.* 5:e1000686 10.1371/journal.pgen.1000686 PMC2757795 19834557

[B57] Moore C. (2012). *“Finding Your Roots with Henry Louis Gates, Jr.” - DNA in the Sixth Episode.* Available at: http://www.yourgeneticgenealogist.com/2012/04/finding-your-roots-with-henry-louis_23.html (accessed 28 June, 2016).

[B58] Mozersky J. Gibbon S. (2014). “Mapping Jewish Identities; migratory histories and the transnational re-framing of’Ashkenazi BRCA mutations’ in the UK and Brazil,” in *Breast Cancer Gene Research and Medical Practices: Transnational Perspectives in the time of BRCA*, eds Nieden A. Z. Palfner S. (New York, NY: Routledge), 35–56. 32134615

[B59] Need A. C. Kasperaviciute D. Cirulli E. T. Goldstein D. B. (2009). A genome-wide genetic signature of Jewish ancestry perfectly separates individuals with and without full Jewish ancestry in a large random sample of European Americans. *Genome Biol.* 10:R7 10.1186/gb-2009-10-1-r7 PMC2687795 19161619

[B60] O’Brien S. J. (1991). Ghetto legacy. *Curr. Biol.* 1 209–211. 10.1016/0960-9822(91)90058-5 15336121

[B61] Ostrer H. (2001). A genetic profile of contemporary Jewish populations. *Nat. Rev. Genet.* 2 891–898. 10.1038/35098506 11715044

[B62] Ostrer H. (2012). *Legacy: A Genetic History of the Jewish People.* Oxford: Oxford University Press.

[B63] Parsons K. J. Cooper W. J. Albertson R. C. (2009). Limits of principal components analysis for producing a common trait space: implications for inferring selection, contingency, and chance in evolution. *PLoS ONE* 4:e7957 10.1371/journal.pone.0007957 PMC2776347 19956767

[B64] Patai R. (1990). *The Hebrew Goddess.* Detroit, MI: Wayne State University Press.

[B65] Patai R. Patai J. (1975). *The Myth of the Jewish Race.* New York, NY: Scribner.

[B66] Purcell S. Neale B. Todd-Brown K. Thomas L. Ferreira M. A. Bender D. (2007). PLINK: a tool set for whole-genome association and population-based linkage analyses. *Am. J. Hum. Genet.* 81 559–575. 10.1086/519795 17701901 PMC1950838

[B67] Ritte U. Neufeld E. Broit M. Shavit D. Motro U. (1993). The differences among Jewish communities—maternal and paternal contributions. *J. Mol. Evol.* 37 435–440. 10.1007/BF00178873 8308911

[B68] Rosenberg S. (2013). *The Jewish Genome Challenge. FailedMessiah.* Available at: http://failedmessiah.typepad.com/failed_messiahcom/2013/09/the-jewish-genome-challenge-345.html (accessed March 21, 2016).

[B69] Sand S. (2009). *The Invention of the Jewish People.* London: Verso.

[B70] Sigal P. (1985). Halakhic perspectives on the matrilineal-patrilineal principles. *Judaism* 34:89.

[B71] Skorecki K. Selig S. Blazer S. Bradman R. Bradman N. Waburton P. J. (1997). Y chromosomes of Jewish priests. *Nature* 385:32 10.1038/385032a0 8985243

[B72] Slepkov N. (2014). *Crowd Sourced Genealogy and Direct-to-Consumer DNA testing: Implications for the Jewish People.* Jerusalem: Jewish People Policy Institute (accessed March 21, 2016).

[B73] Spurdle A. B. Jenkins T. (1996). The origins of the Lemba “Black Jews” of southern Africa: evidence from p12F2 and other Y-chromosome markers. *Am. J. Hum. Genet.* 59 1126–1133. 8900243 PMC1914832

[B74] Tenenbaum S. Davidman L. (2007). It’s in my genes: biological discourse and essentialist views of identity among contemporary American Jews. *Sociol. Q.* 48 435–450. 10.1111/j.1533-8525.2007.00084.x

[B75] Tofanelli S. Ferri G. Bulayeva K. Caciagli L. Onofri V. Taglioli L. (2009). J1-M267 Y lineage marks climate-driven pre-historical human displacements. *Eur. J. Hum. Genet.* 17 1520–1524. 10.1038/ejhg.2009.58 19367321 PMC2986692

[B76] Tofanelli S. Taglioli L. Bertoncini S. Francalacci P. Klyosov A. Pagani L. (2014). Mitochondrial and Y chromosome haplotype motifs as diagnostic markers of Jewish ancestry: a reconsideration. *Front. Genet.* 5:384 10.3389/fgene.2014.00384 PMC4229899 25431579

[B77] van Straten J. Snel H. (2006). The Jewish “demographic miracle” in nineteenth-century Europe fact or fiction? *Hist. Methods* 39 123–131. 10.3200/HMTS.39.3.123-131

[B78] Weiss K. (2015). Are you a “darwinian”?: even academics have totems, but it’s not clear what they imply. *Evol. Anthropol.* 24 43–48. 10.1002/evan.21442 25914357

[B79] Weiss K. M. Long J. C. (2009). Non-Darwinian estimation: my ancestors, my genes’ ancestors. *Genome Res.* 19 703–710. 10.1101/gr.076539.108 19411595 PMC3647532

[B80] Wexler P. (1993). *The Ashkenazic Jews: a Slavo-Turkic People in Search of a Jewish identity.* Colombus, OH: Slavica.

[B81] Yehoshua A. B. (2013). *Defining ‘Who is a Jew’*, Haaretz Available at: http://www.haaretz.com/opinion/.premium-1.545431 (accessed March 21, 2016).

[B82] Zoossmann-Diskin A. (2006). Ashkenazi levites’ “Y modal haplotype” (LMH) – an artificially created phenomenon? *Homo* 57 87–100. 10.1016/j.jchb.2005.12.002 16427053

[B83] Zoossmann-Diskin A. (2010). The origin of Eastern European Jews revealed by autosomal, sex chromosomal and mtDNA polymorphisms. *Biol. Direct.* 5:57 10.1186/1745-6150-5-57 PMC2964539 20925954

