# Supplementary material for: In Search of the jüdische Typus: A Proposed Benchmark to Test the Genetic Basis of Jewishness Challenges Notions of “Jewish Biomarkers”
Source: Front Genet. 2016 Aug 5;7:141. doi: 10.3389/fgene.2016.00141 (PMC4974603; doi:10.3389/fgene.2016.00141)
Supplement: Supplementary file 1 [file Data_Sheet_1.DOCX]

# Supplementary materials

Table S1

Jewish communities and non-Jewish populations included in the benchmark.

| **Sample ID** | **Population** | **Country of origin** |
| --- | --- | --- |
| HGDP01385 | Adygei | Russia, Republic of Adygea |
| HGDP01388 | Adygei | Russia, Republic of Adygea |
| HGDP01381 | Adygei | Russia, Republic of Adygea |
| HGDP01382 | Adygei | Russia, Republic of Adygea |
| HGDP01383 | Adygei | Russia, Republic of Adygea |
| HGDP01387 | Adygei | Russia, Republic of Adygea |
| HGDP01384 | Adygei | Russia, Republic of Adygea |
| HGDP01386 | Adygei | Russia, Republic of Adygea |
| HGDP01396 | Adygei | Russia, Republic of Adygea |
| HGDP01397 | Adygei | Russia, Republic of Adygea |
| HGDP01399 | Adygei | Russia, Republic of Adygea |
| HGDP01400 | Adygei | Russia, Republic of Adygea |
| HGDP01401 | Adygei | Russia, Republic of Adygea |
| HGDP01402 | Adygei | Russia, Republic of Adygea |
| HGDP01398 | Adygei | Russia, Republic of Adygea |
| HGDP01403 | Adygei | Russia, Republic of Adygea |
| HGDP01404 | Adygei | Russia, Republic of Adygea |
| arm3 | Armenian | Armenia |
| arm12 | Armenian | Armenia |
| arm21 | Armenian | Armenia |
| arm4 | Armenian | Armenia |
| arm13 | Armenian | Armenia |
| arm23 | Armenian | Armenia |
| arm5 | Armenian | Armenia |
| arm6 | Armenian | Armenia |
| arm26 | Armenian | Armenia |
| arm7 | Armenian | Armenia |
| arm17 | Armenian | Armenia |
| arm8 | Armenian | Armenia |
| arm14 | Armenian | Armenia |
| arm9 | Armenian | Armenia |
| arm18 | Armenian | Armenia |
| arm10 | Armenian | Armenia |
| arm19 | Armenian | Armenia |
| arm11 | Armenian | Armenia |
| arm20 | Armenian | Armenia |
| GeorgJewtat17 | Azerbaijan Jew | Azerbaijan |
| GeorgJewtat16 | Azerbaijan Jew | Azerbaijan |
| GeorgJewtat2 | Azerbaijan Jew | Azerbaijan |
| GeorgJewtat14 | Azerbaijan Jew | Azerbaijan |
| AzerbJew204 | Azerbaijan Jew | Azerbaijan |
| AzerbJew210 | Azerbaijan Jew | Azerbaijan |
| AzerbJew213 | Azerbaijan Jew | Azerbaijan |
| AzerbJew218 | Azerbaijan Jew | Azerbaijan |
| HGDP00058 | Balochi | Pakistan |
| HGDP00082 | Balochi | Pakistan |
| HGDP00060 | Balochi | Pakistan |
| HGDP00072 | Balochi | Pakistan |
| HGDP00096 | Balochi | Pakistan |
| HGDP00054 | Balochi | Pakistan |
| HGDP00076 | Balochi | Pakistan |
| HGDP00056 | Balochi | Pakistan |
| HGDP00078 | Balochi | Pakistan |
| HGDP00062 | Balochi | Pakistan |
| HGDP00086 | Balochi | Pakistan |
| HGDP00064 | Balochi | Pakistan |
| HGDP00088 | Balochi | Pakistan |
| HGDP00066 | Balochi | Pakistan |
| HGDP00090 | Balochi | Pakistan |
| HGDP00068 | Balochi | Pakistan |
| HGDP00092 | Balochi | Pakistan |
| HGDP00070 | Balochi | Pakistan |
| HGDP00094 | Balochi | Pakistan |
| HGDP00052 | Balochi | Pakistan |
| HGDP00074 | Balochi | Pakistan |
| HGDP00098 | Balochi | Pakistan |
| HGDP00057 | Balochi | Pakistan |
| HGDP00080 | Balochi | Pakistan |
| HGDP01408 | Bantu N.E. | Kenya |
| HGDP01411 | Bantu N.E. | Kenya |
| HGDP01412 | Bantu N.E. | Kenya |
| HGDP01414 | Bantu N.E. | Kenya |
| HGDP01415 | Bantu N.E. | Kenya |
| HGDP01416 | Bantu N.E. | Kenya |
| HGDP01418 | Bantu N.E. | Kenya |
| HGDP01405 | Bantu N.E. | Kenya |
| HGDP01419 | Bantu N.E. | Kenya |
| HGDP01417 | Bantu N.E. | Kenya |
| HGDP01406 | Bantu N.E. | Kenya |
| HGDP00993 | Bantu S.E. Pedi | South Africa |
| HGDP00994 | Bantu S.E. S.Sotho | South Africa |
| HGDP01034 | Bantu S.E. Tswana | South Africa |
| HGDP01030 | Bantu S.E. Tswana | South Africa |
| HGDP01033 | Bantu S.E. Zulu | South Africa |
| HGDP01035 | Bantu S.W. Herero | South Africa |
| HGDP01028 | Bantu S.W. Herero | South Africa |
| HGDP01031 | Bantu S.W. Ovambo | South Africa |
| HGDP00611 | Bedouin | Israel |
| HGDP00623 | Bedouin | Israel |
| HGDP00634 | Bedouin | Israel |
| HGDP00645 | Bedouin | Israel |
| HGDP00612 | Bedouin | Israel |
| HGDP00624 | Bedouin | Israel |
| HGDP00635 | Bedouin | Israel |
| HGDP00646 | Bedouin | Israel |
| HGDP00613 | Bedouin | Israel |
| HGDP00636 | Bedouin | Israel |
| HGDP00647 | Bedouin | Israel |
| HGDP00614 | Bedouin | Israel |
| HGDP00626 | Bedouin | Israel |
| HGDP00637 | Bedouin | Israel |
| HGDP00648 | Bedouin | Israel |
| HGDP00615 | Bedouin | Israel |
| HGDP00627 | Bedouin | Israel |
| HGDP00638 | Bedouin | Israel |
| HGDP00649 | Bedouin | Israel |
| HGDP00616 | Bedouin | Israel |
| HGDP00628 | Bedouin | Israel |
| HGDP00639 | Bedouin | Israel |
| HGDP00650 | Bedouin | Israel |
| HGDP00629 | Bedouin | Israel |
| HGDP00640 | Bedouin | Israel |
| HGDP00651 | Bedouin | Israel |
| HGDP00607 | Bedouin | Israel |
| HGDP00619 | Bedouin | Israel |
| HGDP00631 | Bedouin | Israel |
| HGDP00642 | Bedouin | Israel |
| HGDP00653 | Bedouin | Israel |
| HGDP00641 | Bedouin | Israel |
| HGDP00643 | Bedouin | Israel |
| HGDP00654 | Bedouin | Israel |
| HGDP00644 | Bedouin | Israel |
| HGDP00701 | Bedouin | Israel |
| HGDP00610 | Bedouin | Israel |
| HGDP00622 | Bedouin | Israel |
| HGDP00625 | Bedouin | Israel |
| HGDP00608 | Bedouin | Israel |
| HGDP00620 | Bedouin | Israel |
| HGDP00632 | Bedouin | Israel |
| HGDP00618 | Bedouin | Israel |
| HGDP00630 | Bedouin | Israel |
| HGDP00609 | Bedouin | Israel |
| belorus1 | Belarusian | Belarus |
| belorus2 | Belarusian | Belarus |
| belorus3 | Belarusian | Belarus |
| belorus4 | Belarusian | Belarus |
| belorus5 | Belarusian | Belarus |
| belorus6 | Belarusian | Belarus |
| belorus7 | Belarusian | Belarus |
| belorus8 | Belarusian | Belarus |
| belorus10 | Belarusian | Belarus |
| HGDP00479 | Biaka Pygmy | Central African Republic |
| HGDP00985 | Biaka Pygmy | Central African Republic |
| HGDP01094 | Biaka Pygmy | Central African Republic |
| HGDP00461 | Biaka Pygmy | Central African Republic |
| HGDP00986 | Biaka Pygmy | Central African Republic |
| HGDP00464 | Biaka Pygmy | Central African Republic |
| HGDP00465 | Biaka Pygmy | Central African Republic |
| HGDP00466 | Biaka Pygmy | Central African Republic |
| HGDP00454 | Biaka Pygmy | Central African Republic |
| HGDP01086 | Biaka Pygmy | Central African Republic |
| HGDP00469 | Biaka Pygmy | Central African Republic |
| HGDP00455 | Biaka Pygmy | Central African Republic |
| HGDP01087 | Biaka Pygmy | Central African Republic |
| HGDP00470 | Biaka Pygmy | Central African Republic |
| HGDP00457 | Biaka Pygmy | Central African Republic |
| HGDP00473 | Biaka Pygmy | Central African Republic |
| HGDP00459 | Biaka Pygmy | Central African Republic |
| HGDP01090 | Biaka Pygmy | Central African Republic |
| HGDP00472 | Biaka Pygmy | Central African Republic |
| HGDP00458 | Biaka Pygmy | Central African Republic |
| HGDP00475 | Biaka Pygmy | Central African Republic |
| HGDP00460 | Biaka Pygmy | Central African Republic |
| Indian_Jew_5 | Bnei Israel Jew | India, Mumbai |
| Indian_Jew_6 | Bnei Israel Jew | India, Mumbai |
| Indian_Jew_7 | Bnei Israel Jew | India, Mumbai |
| Indian_Jew_8 | Bnei Israel Jew | India, Mumbai |
| HGDP00011 | Brahui | Pakistan |
| HGDP00035 | Brahui | Pakistan |
| HGDP00013 | Brahui | Pakistan |
| HGDP00037 | Brahui | Pakistan |
| HGDP00001 | Brahui | Pakistan |
| HGDP00025 | Brahui | Pakistan |
| HGDP00049 | Brahui | Pakistan |
| HGDP00005 | Brahui | Pakistan |
| HGDP00029 | Brahui | Pakistan |
| HGDP00007 | Brahui | Pakistan |
| HGDP00031 | Brahui | Pakistan |
| HGDP00015 | Brahui | Pakistan |
| HGDP00039 | Brahui | Pakistan |
| HGDP00017 | Brahui | Pakistan |
| HGDP00041 | Brahui | Pakistan |
| HGDP00019 | Brahui | Pakistan |
| HGDP00043 | Brahui | Pakistan |
| HGDP00021 | Brahui | Pakistan |
| HGDP00045 | Brahui | Pakistan |
| HGDP00009 | Brahui | Pakistan |
| HGDP00047 | Brahui | Pakistan |
| HGDP00003 | Brahui | Pakistan |
| HGDP00027 | Brahui | Pakistan |
| HGDP00033 | Brahui | Pakistan |
| HGDP00023 | Brahui | Pakistan |
| HGDP00356 | Burusho | Pakistan |
| HGDP00412 | Burusho | Pakistan |
| HGDP00359 | Burusho | Pakistan |
| HGDP00417 | Burusho | Pakistan |
| HGDP00364 | Burusho | Pakistan |
| HGDP00423 | Burusho | Pakistan |
| HGDP00371 | Burusho | Pakistan |
| HGDP00428 | Burusho | Pakistan |
| HGDP00376 | Burusho | Pakistan |
| HGDP00438 | Burusho | Pakistan |
| HGDP00341 | Burusho | Pakistan |
| HGDP00397 | Burusho | Pakistan |
| HGDP00402 | Burusho | Pakistan |
| HGDP00351 | Burusho | Pakistan |
| HGDP00407 | Burusho | Pakistan |
| HGDP00372 | Burusho | Pakistan |
| HGDP00433 | Burusho | Pakistan |
| HGDP00382 | Burusho | Pakistan |
| HGDP00444 | Burusho | Pakistan |
| HGDP00336 | Burusho | Pakistan |
| HGDP00388 | Burusho | Pakistan |
| HGDP00445 | Burusho | Pakistan |
| HGDP00338 | Burusho | Pakistan |
| HGDP00392 | Burusho | Pakistan |
| HGDP00346 | Burusho | Pakistan |
| HGDP00712 | Cambodian | Cambodia |
| HGDP00713 | Cambodian | Cambodia |
| HGDP00714 | Cambodian | Cambodia |
| HGDP00715 | Cambodian | Cambodia |
| HGDP00716 | Cambodian | Cambodia |
| HGDP00717 | Cambodian | Cambodia |
| HGDP00720 | Cambodian | Cambodia |
| HGDP00711 | Cambodian | Cambodia |
| HGDP00721 | Cambodian | Cambodia |
| HGDP00719 | Cambodian | Cambodia |
| ashkenazy1w | Ashkenazic Jew | Austria |
| ashkenazy2w | Ashkenazic Jew | Austria |
| ashkenazy3w | Ashkenazic Jew | Austria |
| ashkenazy4w | Ashkenazic Jew | France |
| ashkenazy6w | Ashkenazic Jew | Germany |
| ashkenazy7w | Ashkenazic Jew | Germany |
| ashkenazy8w | Ashkenazic Jew | Netherlands |
| ashkenazy9w | Ashkenazic Jew | Netherlands |
| ashkenazy10w | Ashkenazic Jew | Netherlands |
| chuvash1 | Chuvash | Russia |
| chuvash2 | Chuvash | Russia |
| chuvash3 | Chuvash | Russia |
| chuvash4 | Chuvash | Russia |
| chuvash5 | Chuvash | Russia |
| chuvash6 | Chuvash | Russia |
| chuvash7 | Chuvash | Russia |
| chuvash8 | Chuvash | Russia |
| chuvash9 | Chuvash | Russia |
| chuvash10 | Chuvash | Russia |
| chuvash11 | Chuvash | Russia |
| chuvash12 | Chuvash | Russia |
| chuvash13 | Chuvash | Russia |
| chuvash14 | Chuvash | Russia |
| chuvash15 | Chuvash | Russia |
| chuvash16 | Chuvash | Russia |
| chuvash17 | Chuvash | Russia |
| Indian_Jew_1 | Cochin Jew | India, Cochin |
| Indian_Jew_2 | Cochin Jew | India, Cochin |
| Indian_Jew_3 | Cochin Jew | India, Cochin |
| Indian_Jew_4 | Cochin Jew | India, Cochin |
| Cyprus1 | Cypriot | Cyprus |
| Cyprus2 | Cypriot | Cyprus |
| Cyprus3 | Cypriot | Cyprus |
| Cyprus4 | Cypriot | Cyprus |
| Cyprus5 | Cypriot | Cyprus |
| Cyprus6 | Cypriot | Cyprus |
| Cyprus7 | Cypriot | Cyprus |
| Cyprus8 | Cypriot | Cyprus |
| Cyprus9 | Cypriot | Cyprus |
| Cyprus10 | Cypriot | Cyprus |
| Cyprus11 | Cypriot | Cyprus |
| Cyprus12 | Cypriot | Cyprus |
| HGDP01308 | Dai | China |
| HGDP01310 | Dai | China |
| HGDP01311 | Dai | China |
| HGDP01307 | Dai | China |
| HGDP01309 | Dai | China |
| HGDP01312 | Dai | China |
| HGDP01313 | Dai | China |
| HGDP01314 | Dai | China |
| HGDP01315 | Dai | China |
| HGDP01316 | Dai | China |
| HGDP01213 | Daur | China |
| HGDP01214 | Daur | China |
| HGDP01221 | Daur | China |
| HGDP01222 | Daur | China |
| HGDP01216 | Daur | China |
| HGDP01218 | Daur | China |
| HGDP01215 | Daur | China |
| HGDP01217 | Daur | China |
| HGDP01220 | Daur | China |
| HGDP00557 | Druze | Israel |
| HGDP00569 | Druze | Israel |
| HGDP00581 | Druze | Israel |
| HGDP00594 | Druze | Israel |
| HGDP00558 | Druze | Israel |
| HGDP00582 | Druze | Israel |
| HGDP00595 | Druze | Israel |
| HGDP00559 | Druze | Israel |
| HGDP00571 | Druze | Israel |
| HGDP00583 | Druze | Israel |
| HGDP00597 | Druze | Israel |
| HGDP00572 | Druze | Israel |
| HGDP00584 | Druze | Israel |
| HGDP00598 | Druze | Israel |
| HGDP00561 | Druze | Israel |
| HGDP00573 | Druze | Israel |
| HGDP00599 | Druze | Israel |
| HGDP00562 | Druze | Israel |
| HGDP00574 | Druze | Israel |
| HGDP00586 | Druze | Israel |
| HGDP00600 | Druze | Israel |
| HGDP00563 | Druze | Israel |
| HGDP00575 | Druze | Israel |
| HGDP00587 | Druze | Israel |
| HGDP00601 | Druze | Israel |
| HGDP00565 | Druze | Israel |
| HGDP00577 | Druze | Israel |
| HGDP00564 | Druze | Israel |
| HGDP00576 | Druze | Israel |
| HGDP00588 | Druze | Israel |
| HGDP00602 | Druze | Israel |
| HGDP00566 | Druze | Israel |
| HGDP00578 | Druze | Israel |
| HGDP00590 | Druze | Israel |
| HGDP00604 | Druze | Israel |
| HGDP00567 | Druze | Israel |
| HGDP00579 | Druze | Israel |
| HGDP00591 | Druze | Israel |
| HGDP00560 | Druze | Israel |
| HGDP00568 | Druze | Israel |
| HGDP00580 | Druze | Israel |
| HGDP00606 | Druze | Israel |
| ashkenazy1e | Ashkenazic Jew | Belarus |
| ashkenazy2e | Ashkenazic Jew | Belarus |
| ashkenazy3e | Ashkenazic Jew | Latvia |
| ashkenazy4e | Ashkenazic Jew | Latvia |
| ashkenazy5e | Ashkenazic Jew | Lithuania |
| ashkenazy7e | Ashkenazic Jew | Poland |
| ashkenazy8e | Ashkenazic Jew | Poland |
| ashkenazy9e | Ashkenazic Jew | Poland |
| ashkenazy10e | Ashkenazic Jew | Russia |
| Romanian_Jew_1 | Ashkenazic Jew | Romania |
| Romanian_Jew_2 | Ashkenazic Jew | Romania |
| Romanian_Jew_3 | Ashkenazic Jew | Romania |
| Egypt1 | Egyptian | Egypt |
| Egypt2 | Egyptians | Egypt |
| Egypt3 | Egyptian | Egypt |
| Egypt4 | Egyptian | Egypt |
| Egypt5 | Egyptian | Egypt |
| Egypt6 | Egyptian | Egypt |
| Egypt7 | Egyptian | Egypt |
| Egypt8 | Egyptian | Egypt |
| Egypt9 | Egyptian | Egypt |
| Egypt10 | Egyptian | Egypt |
| Egypt11 | Egyptian | Egypt |
| Egypt12 | Egyptian | Egypt |
| eth_jew8 | Ethiopian Jew | Ethiopia |
| eth_jew10 | Ethiopian Jew | Ethiopia |
| eth_jew11 | Ethiopian Jew | Ethiopia |
| eth_jew12 | Ethiopian Jew | Ethiopia |
| eth_jew13 | Ethiopian Jew | Ethiopia |
| eth_jew14 | Ethiopian Jew | Ethiopia |
| eth_jew1 | Ethiopian Jew | Ethiopia |
| eth_jew2 | Ethiopian Jew | Ethiopia |
| eth_jew3 | Ethiopian Jew | Ethiopia |
| eth_jew4 | Ethiopian Jew | Ethiopia |
| eth_jew5 | Ethiopian Jew | Ethiopia |
| eth_jew6 | Ethiopian Jew | Ethiopia |
| eth_jew7 | Ethiopian Jew | Ethiopia |
| EthiopiaAm52 | Ethiopians A | Ethiopia |
| EthiopiaAm50 | Ethiopians A | Ethiopia |
| EthiopiaAm58 | Ethiopians A | Ethiopia |
| EthiopiaAm59 | Ethiopians A | Ethiopia |
| EthiopiaAm76 | Ethiopians A | Ethiopia |
| EthiopiaAm77 | Ethiopians A | Ethiopia |
| EthiopiaAm78 | Ethiopians A | Ethiopia |
| EthiopiaOr41c | Ethiopians O | Ethiopia |
| EthiopiaOr42a | Ethiopians O | Ethiopia |
| EthiopiaOr44b | Ethiopians O | Ethiopia |
| EthiopiaOr45a | Ethiopians O | Ethiopia |
| EthiopiaOr48c | Ethiopians O | Ethiopia |
| EthiopiaOr53b | Ethiopians O | Ethiopia |
| EthiopiaOr48 | Ethiopians O | Ethiopia |
| EthiopiaTi5 | Ethiopians T | Ethiopia |
| EthiopiaTi7 | Ethiopians T | Ethiopia |
| EthiopiaTi61 | Ethiopians T | Ethiopia |
| EthiopiaTi63 | Ethiopians T | Ethiopia |
| EthiopiaTi69 | Ethiopians T | Ethiopia |
| HGDP00521 | French | France |
| HGDP00533 | French | France |
| HGDP00522 | French | France |
| HGDP00534 | French | France |
| HGDP00511 | French | France |
| HGDP00523 | French | France |
| HGDP00535 | French | France |
| HGDP00512 | French | France |
| HGDP00524 | French | France |
| HGDP00536 | French | France |
| HGDP00513 | French | France |
| HGDP00525 | French | France |
| HGDP00537 | French | France |
| HGDP00514 | French | France |
| HGDP00526 | French | France |
| HGDP00538 | French | France |
| HGDP00515 | French | France |
| HGDP00527 | French | France |
| HGDP00539 | French | France |
| HGDP00520 | French | France |
| HGDP00517 | French | France |
| HGDP00529 | French | France |
| HGDP00519 | French | France |
| HGDP00531 | French | France |
| HGDP00518 | French | France |
| HGDP00530 | French | France |
| HGDP00528 | French | France |
| HGDP00516 | French | France |
| HGDP01361 | French Basque | France |
| HGDP01372 | French Basque | France |
| HGDP01362 | French Basque | France |
| HGDP01373 | French Basque | France |
| HGDP01363 | French Basque | France |
| HGDP01374 | French Basque | France |
| HGDP01370 | French Basque | France |
| HGDP01366 | French Basque | France |
| HGDP01377 | French Basque | France |
| HGDP01358 | French Basque | France |
| HGDP01360 | French Basque | France |
| HGDP01367 | French Basque | France |
| HGDP01378 | French Basque | France |
| HGDP01368 | French Basque | France |
| HGDP01379 | French Basque | France |
| HGDP01369 | French Basque | France |
| HGDP01380 | French Basque | France |
| HGDP01371 | French Basque | France |
| HGDP01359 | French Basque | France |
| HGDP01364 | French Basque | France |
| HGDP01375 | French Basque | France |
| HGDP01365 | French Basque | France |
| HGDP01357 | French Basque | France |
| HGDP01376 | French Basque | France |
| mg65 | Georgia | Caucasus |
| mg20 | Georgia | Caucasus |
| mg43 | Georgia | Caucasus |
| mg5 | Georgia | Caucasus |
| mg34 | Georgia | Caucasus |
| mg68 | Georgia | Caucasus |
| mg54 | Georgia | Caucasus |
| mg64 | Georgia | Caucasus |
| mg22 | Georgia | Caucasus |
| mg47 | Georgia | Caucasus |
| mg27 | Georgia | Caucasus |
| mg23 | Georgia | Caucasus |
| mg49 | Georgia | Caucasus |
| mg40 | Georgia | Caucasus |
| mg51 | Georgia | Caucasus |
| mg62 | Georgia | Caucasus |
| mg31 | Georgia | Caucasus |
| mg61 | Georgia | Caucasus |
| mg70 | Georgia | Caucasus |
| mg72 | Georgia | Caucasus |
| GeorgJew105 | Georgia Jew | Georgia |
| GeorgJew111 | Georgia Jew | Georgia |
| GeorgJew125 | Georgia Jew | Georgia |
| GeorgJew141 | Georgia Jew | Georgia |
| HGDP00973 | Han | China |
| HGDP00777 | Han | China |
| HGDP00820 | Han | China |
| HGDP01291 | Han | China |
| HGDP01021 | Han | China |
| HGDP00778 | Han | China |
| HGDP00821 | Han | China |
| HGDP01292 | Han | China |
| HGDP00775 | Han | China |
| HGDP00780 | Han | China |
| HGDP00822 | Han | China |
| HGDP01293 | Han | China |
| HGDP00779 | Han | China |
| HGDP00781 | Han | China |
| HGDP00972 | Han | China |
| HGDP01294 | Han | China |
| HGDP00815 | Han | China |
| HGDP00785 | Han | China |
| HGDP00976 | Han | China |
| HGDP01296 | Han | China |
| HGDP00782 | Han | China |
| HGDP00813 | Han | China |
| HGDP01288 | Han | China |
| HGDP00783 | Han | China |
| HGDP00774 | Han | China |
| HGDP00817 | Han | China |
| HGDP01289 | Han | China |
| HGDP00971 | Han | China |
| HGDP00776 | Han | China |
| HGDP00819 | Han | China |
| HGDP01290 | Han | China |
| HGDP00814 | Han | China |
| HGDP00784 | Han | China |
| HGDP00975 | Han | China |
| HGDP01295 | Han | China |
| HGDP00818 | Han | China |
| HGDP00786 | Han | China |
| HGDP00977 | Han | China |
| HGDP00974 | Han | China |
| HGDP00811 | Han | China |
| HGDP01023 | Han | China |
| HGDP00812 | Han | China |
| HGDP01287 | Han | China |
| HGDP01024 | Han | China |
| HGDP00103 | Hazara | Pakistan |
| HGDP00104 | Hazara | Pakistan |
| HGDP00099 | Hazara | Pakistan |
| HGDP00100 | Hazara | Pakistan |
| HGDP00105 | Hazara | Pakistan |
| HGDP00106 | Hazara | Pakistan |
| HGDP00119 | Hazara | Pakistan |
| HGDP00108 | Hazara | Pakistan |
| HGDP00120 | Hazara | Pakistan |
| HGDP00109 | Hazara | Pakistan |
| HGDP00121 | Hazara | Pakistan |
| HGDP00110 | Hazara | Pakistan |
| HGDP00122 | Hazara | Pakistan |
| HGDP00102 | Hazara | Pakistan |
| HGDP00112 | Hazara | Pakistan |
| HGDP00125 | Hazara | Pakistan |
| HGDP00115 | Hazara | Pakistan |
| HGDP00129 | Hazara | Pakistan |
| HGDP00116 | Hazara | Pakistan |
| HGDP00118 | Hazara | Pakistan |
| HGDP00124 | Hazara | Pakistan |
| HGDP00127 | Hazara | Pakistan |
| HGDP01237 | Hezhen | China |
| HGDP01238 | Hezhen | China |
| HGDP01236 | Hezhen | China |
| HGDP01234 | Hezhen | China |
| HGDP01235 | Hezhen | China |
| HGDP01240 | Hezhen | China |
| HGDP01242 | Hezhen | China |
| HGDP01239 | Hezhen | China |
| HGDP01241 | Hezhen | China |
| hungary1 | Hungarian | Hungary |
| hungary2 | Hungarian | Hungary |
| hungary3 | Hungarian | Hungary |
| hungary4 | Hungarian | Hungary |
| hungary5 | Hungarian | Hungary |
| hungary6 | Hungarian | Hungary |
| hungary7 | Hungarian | Hungary |
| hungary8 | Hungarian | Hungary |
| hungary9 | Hungarian | Hungary |
| hungary10 | Hungarian | Hungary |
| hungary11 | Hungarian | Hungary |
| hungary12 | Hungarian | Hungary |
| hungary13 | Hungarian | Hungary |
| hungary14 | Hungarian | Hungary |
| hungary15 | Hungarian | Hungary |
| hungary16 | Hungarian | Hungary |
| hungary17 | Hungarian | Hungary |
| hungary18 | Hungarian | Hungary |
| hungary19 | Hungarian | Hungary |
| hungary20 | Hungarian | Hungary |
| iran_jew1 | Iranian Jew | Iran |
| iran_jew2 | Iranian Jew | Iran |
| iran_jew3 | Iranian Jew | Iran |
| iran_jew4 | Iranian Jew | Iran |
| iran1 | Iranian | Iran |
| iran2 | Iranian | Iran |
| iran3 | Iranian | Iran |
| iran4 | Iranian | Iran |
| iran5 | Iranian | Iran |
| iran6 | Iranian | Iran |
| iran7 | Iranian | Iran |
| iran8 | Iranian | Iran |
| iran9 | Iranian | Iran |
| iran10 | Iranian | Iran |
| iran11 | Iranian | Iran |
| iran12 | Iranian | Iran |
| iran13 | Iranian | Iran |
| iran14 | Iranian | Iran |
| iran15 | Iranian | Iran |
| iran16 | Iranian | Iran |
| iran17 | Iranian | Iran |
| iran18 | Iranian | Iran |
| iran19 | Iranian | Iran |
| iran20 | Iranian | Iran |
| iraq_jew1 | Iraq Jew | Iraq |
| iraq_jew2 | Iraq Jew | Iraq |
| iraq_jew3 | Iraq Jew | Iraq |
| iraq_jew4 | Iraq Jew | Iraq |
| iraq_jew5 | Iraq Jew | Iraq |
| iraq_jew6 | Iraq Jew | Iraq |
| iraq_jew7 | Iraq Jew | Iraq |
| iraq_jew8 | Iraq Jew | Iraq |
| iraq_jew9 | Iraq Jew | Iraq |
| iraq_jew10 | Iraq Jew | Iraq |
| iraq_jew11 | Iraq Jew | Iraq |
| HGDP00747 | Japanese | Japan |
| HGDP00759 | Japanese | Japan |
| HGDP00769 | Japanese | Japan |
| HGDP00748 | Japanese | Japan |
| HGDP00760 | Japanese | Japan |
| HGDP00749 | Japanese | Japan |
| HGDP00761 | Japanese | Japan |
| HGDP00771 | Japanese | Japan |
| HGDP00750 | Japanese | Japan |
| HGDP00762 | Japanese | Japan |
| HGDP00772 | Japanese | Japan |
| HGDP00751 | Japanese | Japan |
| HGDP00763 | Japanese | Japan |
| HGDP00773 | Japanese | Japan |
| HGDP00758 | Japanese | Japan |
| HGDP00752 | Japanese | Japan |
| HGDP00764 | Japanese | Japan |
| HGDP00790 | Japanese | Japan |
| HGDP00753 | Japanese | Japan |
| HGDP00765 | Japanese | Japan |
| HGDP00791 | Japanese | Japan |
| HGDP00756 | Japanese | Japan |
| HGDP00755 | Japanese | Japan |
| HGDP00767 | Japanese | Japan |
| HGDP00757 | Japanese | Japan |
| HGDP00768 | Japanese | Japan |
| HGDP00828 | Japanese | Japan |
| HGDP00766 | Japanese | Japan |
| Jordan444 | Jordanian | Jordan |
| Jordan608 | Jordanian | Jordan |
| Jordan307 | Jordanian | Jordan |
| Jordan646 | Jordanian | Jordan |
| Jordan543 | Jordanian | Jordan |
| Jordan214 | Jordanian | Jordan |
| Jordan503 | Jordanian | Jordan |
| Jordan384 | Jordanian | Jordan |
| Jordan305 | Jordanian | Jordan |
| Jordan445 | Jordanian | Jordan |
| Jordan387 | Jordanian | Jordan |
| Jordan62 | Jordanian | Jordan |
| Jordan382 | Jordanian | Jordan |
| Jordan603 | Jordanian | Jordan |
| Jordan426 | Jordanian | Jordan |
| Jordan502 | Jordanian | Jordan |
| Jordan485 | Jordanian | Jordan |
| Jordan546 | Jordanian | Jordan |
| Jordan563 | Jordanian | Jordan |
| Jordan560 | Jordanian | Jordan |
| HGDP01323 | Lahu | China |
| HGDP01326 | Lahu | China |
| HGDP01317 | Lahu | China |
| HGDP01318 | Lahu | China |
| HGDP01321 | Lahu | China |
| HGDP01320 | Lahu | China |
| HGDP01322 | Lahu | China |
| HGDP01319 | Lahu | China |
| Lebanon2 | Lebanese | Lebanon |
| Lebanon3 | Lebanese | Lebanon |
| Lebanon4 | Lebanese | Lebanon |
| Lebanon5 | Lebanese | Lebanon |
| Lebanon6 | Lebanese | Lebanon |
| Lebanon7 | Lebanese | Lebanon |
| Lebanon8 | Lebanese | Lebanon |
| lez7 | Lezgin | Dagestan |
| lez9 | Lezgin | Dagestan |
| lez13 | Lezgin | Dagestan |
| lez15 | Lezgin | Dagestan |
| lez17 | Lezgin | Dagestan |
| lez3 | Lezgin | Dagestan |
| lez24 | Lezgin | Dagestan |
| lez25 | Lezgin | Dagestan |
| lez44 | Lezgin | Dagestan |
| lez31 | Lezgin | Dagestan |
| lez49 | Lezgin | Dagestan |
| lez33 | Lezgin | Dagestan |
| lez52 | Lezgin | Dagestan |
| lez36 | Lezgin | Dagestan |
| lez37 | Lezgin | Dagestan |
| lez38 | Lezgin | Dagestan |
| lez40 | Lezgin | Dagestan |
| lez42 | Lezgin | Dagestan |
| lithuania1 | Lithuanian | Lithuania |
| lithuania2 | Lithuanian | Lithuania |
| lithuania3 | Lithuanian | Lithuania |
| lithuania4 | Lithuanian | Lithuania |
| lithuania5 | Lithuanian | Lithuania |
| lithuania6 | Lithuanian | Lithuania |
| lithuania7 | Lithuanian | Lithuania |
| lithuania8 | Lithuanian | Lithuania |
| lithuania9 | Lithuanian | Lithuania |
| lithuania10 | Lithuanian | Lithuania |
| HGDP00131 | Makrani | Pakistan |
| HGDP00146 | Makrani | Pakistan |
| HGDP00133 | Makrani | Pakistan |
| HGDP00148 | Makrani | Pakistan |
| HGDP00134 | Makrani | Pakistan |
| HGDP00149 | Makrani | Pakistan |
| HGDP00135 | Makrani | Pakistan |
| HGDP00150 | Makrani | Pakistan |
| HGDP00137 | Makrani | Pakistan |
| HGDP00153 | Makrani | Pakistan |
| HGDP00143 | Makrani | Pakistan |
| HGDP00158 | Makrani | Pakistan |
| HGDP00130 | Makrani | Pakistan |
| HGDP00144 | Makrani | Pakistan |
| HGDP00160 | Makrani | Pakistan |
| HGDP00145 | Makrani | Pakistan |
| HGDP00161 | Makrani | Pakistan |
| HGDP00136 | Makrani | Pakistan |
| HGDP00151 | Makrani | Pakistan |
| HGDP00139 | Makrani | Pakistan |
| HGDP00154 | Makrani | Pakistan |
| HGDP00140 | Makrani | Pakistan |
| HGDP00155 | Makrani | Pakistan |
| HGDP00141 | Makrani | Pakistan |
| HGDP00157 | Makrani | Pakistan |
| HGDP00911 | Mandenka | Senegal |
| HGDP01202 | Mandenka | Senegal |
| HGDP00912 | Mandenka | Senegal |
| HGDP01283 | Mandenka | Senegal |
| HGDP00913 | Mandenka | Senegal |
| HGDP01284 | Mandenka | Senegal |
| HGDP00914 | Mandenka | Senegal |
| HGDP01285 | Mandenka | Senegal |
| HGDP00915 | Mandenka | Senegal |
| HGDP01286 | Mandenka | Senegal |
| HGDP00904 | Mandenka | Senegal |
| HGDP00905 | Mandenka | Senegal |
| HGDP00917 | Mandenka | Senegal |
| HGDP00907 | Mandenka | Senegal |
| HGDP00906 | Mandenka | Senegal |
| HGDP00918 | Mandenka | Senegal |
| HGDP00908 | Mandenka | Senegal |
| HGDP01199 | Mandenka | Senegal |
| HGDP00909 | Mandenka | Senegal |
| HGDP01200 | Mandenka | Senegal |
| HGDP00910 | Mandenka | Senegal |
| HGDP01201 | Mandenka | Senegal |
| HGDP00982 | Mbuti Pygmy | Democratic Republic of Congo |
| HGDP00449 | Mbuti Pygmy | Democratic Republic of Congo |
| HGDP00450 | Mbuti Pygmy | Democratic Republic of Congo |
| HGDP00984 | Mbuti Pygmy | Democratic Republic of Congo |
| HGDP00456 | Mbuti Pygmy | Democratic Republic of Congo |
| HGDP01081 | Mbuti Pygmy | Democratic Republic of Congo |
| HGDP00462 | Mbuti Pygmy | Democratic Republic of Congo |
| HGDP00463 | Mbuti Pygmy | Democratic Republic of Congo |
| HGDP00467 | Mbuti Pygmy | Democratic Republic of Congo |
| HGDP00471 | Mbuti Pygmy | Democratic Republic of Congo |
| HGDP00474 | Mbuti Pygmy | Democratic Republic of Congo |
| HGDP00476 | Mbuti Pygmy | Democratic Republic of Congo |
| HGDP00478 | Mbuti Pygmy | Democratic Republic of Congo |
| HGDP01198 | Miaozu | China |
| HGDP01197 | Miaozu | China |
| HGDP01194 | Miaozu | China |
| HGDP01195 | Miaozu | China |
| HGDP01196 | Miaozu | China |
| HGDP01189 | Miaozu | China |
| HGDP01191 | Miaozu | China |
| HGDP01193 | Miaozu | China |
| HGDP01192 | Miaozu | China |
| HGDP01190 | Miaozu | China |
| HGDP01225 | Mongola | China |
| HGDP01226 | Mongola | China |
| HGDP01224 | Mongola | China |
| HGDP01223 | Mongola | China |
| HGDP01228 | Mongola | China |
| HGDP01230 | Mongola | China |
| HGDP01231 | Mongola | China |
| HGDP01227 | Mongola | China |
| HGDP01229 | Mongola | China |
| HGDP01232 | Mongola | China |
| mongol107 | Mongol | Mongolia |
| mongol114 | Mongol | Mongolia |
| mongol115 | Mongol | Mongolia |
| mongol119 | Mongol | Mongolia |
| mongol120 | Mongol | Mongolia |
| mongol123 | Mongol | Mongolia |
| mongol124 | Mongol | Mongolia |
| mongol128 | Mongol | Mongolia |
| mongol131 | Mongol | Mongolia |
| moroccoA98 | Moroccan | Morocco |
| moroccoB93 | Moroccan | Morocco |
| moroccoA57 | Moroccan | Morocco |
| moroccoA121 | Moroccan | Morocco |
| moroccoA62 | Moroccan | Morocco |
| moroccoC61 | Moroccan | Morocco |
| moroccoE28 | Moroccan | Morocco |
| moroccoE21 | Moroccan | Morocco |
| moroccoC60 | Moroccan | Morocco |
| moroccoB17 | Moroccan | Morocco |
| Morocco_Jew_1 | Morocco Jew | Morocco |
| Morocco_Jew_2 | Morocco Jew | Morocco |
| Morocco_Jew_3 | Morocco Jew | Morocco |
| Morocco_Jew_4 | Morocco Jew | Morocco |
| Morocco_Jew_5 | Morocco Jew | Morocco |
| Morocco_Jew_6 | Morocco Jew | Morocco |
| Morocco_Jew_7 | Morocco Jew | Morocco |
| Morocco_Jew_8 | Morocco Jew | Morocco |
| Morocco_Jew_9 | Morocco Jew | Morocco |
| Morocco_Jew_10 | Morocco Jew | Morocco |
| Morocco_Jew_11 | Morocco Jew | Morocco |
| Morocco_Jew_12 | Morocco Jew | Morocco |
| Morocco_Jew_13 | Morocco Jew | Morocco |
| Morocco_Jew_14 | Morocco Jew | Morocco |
| Morocco_Jew_15 | Morocco Jew | Morocco |
| MorJew2001 | Morocco Jew | Morocco |
| HGDP01263 | Mozabite | Algeria (Mzab) |
| HGDP01275 | Mozabite | Algeria (Mzab) |
| HGDP01264 | Mozabite | Algeria (Mzab) |
| HGDP01276 | Mozabite | Algeria (Mzab) |
| HGDP01253 | Mozabite | Algeria (Mzab) |
| HGDP01265 | Mozabite | Algeria (Mzab) |
| HGDP01277 | Mozabite | Algeria (Mzab) |
| HGDP01254 | Mozabite | Algeria (Mzab) |
| HGDP01266 | Mozabite | Algeria (Mzab) |
| HGDP01278 | Mozabite | Algeria (Mzab) |
| HGDP01255 | Mozabite | Algeria (Mzab) |
| HGDP01267 | Mozabite | Algeria (Mzab) |
| HGDP01279 | Mozabite | Algeria (Mzab) |
| HGDP01256 | Mozabite | Algeria (Mzab) |
| HGDP01268 | Mozabite | Algeria (Mzab) |
| HGDP01280 | Mozabite | Algeria (Mzab) |
| HGDP01257 | Mozabite | Algeria (Mzab) |
| HGDP01269 | Mozabite | Algeria (Mzab) |
| HGDP01259 | Mozabite | Algeria (Mzab) |
| HGDP01262 | Mozabite | Algeria (Mzab) |
| HGDP01274 | Mozabite | Algeria (Mzab) |
| HGDP01260 | Mozabite | Algeria (Mzab) |
| HGDP01272 | Mozabite | Algeria (Mzab) |
| HGDP01258 | Mozabite | Algeria (Mzab) |
| HGDP01282 | Mozabite | Algeria (Mzab) |
| HGDP01261 | Mozabite | Algeria (Mzab) |
| HGDP01273 | Mozabite | Algeria (Mzab) |
| HGDP01337 | Naxi | China |
| HGDP01338 | Naxi | China |
| HGDP01339 | Naxi | China |
| HGDP01346 | Naxi | China |
| HGDP01340 | Naxi | China |
| HGDP01341 | Naxi | China |
| HGDP01345 | Naxi | China |
| HGDP01342 | Naxi | China |
| HGDP00794 | Orcadian | United Kingdom |
| HGDP00806 | Orcadian | United Kingdom |
| HGDP00795 | Orcadian | United Kingdom |
| HGDP00807 | Orcadian | United Kingdom |
| HGDP00796 | Orcadian | United Kingdom |
| HGDP00808 | Orcadian | United Kingdom |
| HGDP00803 | Orcadian | United Kingdom |
| HGDP00799 | Orcadian | United Kingdom |
| HGDP00800 | Orcadian | United Kingdom |
| HGDP00805 | Orcadian | United Kingdom |
| HGDP00802 | Orcadian | United Kingdom |
| HGDP00804 | Orcadian | United Kingdom |
| HGDP00797 | Orcadian | United Kingdom |
| HGDP00810 | Orcadian | United Kingdom |
| HGDP00798 | Orcadian | United Kingdom |
| HGDP01212 | Oroqen | China |
| HGDP01209 | Oroqen | China |
| HGDP01211 | Oroqen | China |
| HGDP01204 | Oroqen | China |
| HGDP01206 | Oroqen | China |
| HGDP01208 | Oroqen | China |
| HGDP01207 | Oroqen | China |
| HGDP01203 | Oroqen | China |
| HGDP01205 | Oroqen | China |
| HGDP00675 | Palestinian | Israel |
| HGDP00687 | Palestinian | Israel |
| HGDP00697 | Palestinian | Israel |
| HGDP00729 | Palestinian | Israel |
| HGDP00741 | Palestinian | Israel |
| HGDP00676 | Palestinian | Israel |
| HGDP00688 | Palestinian | Israel |
| HGDP00698 | Palestinian | Israel |
| HGDP00730 | Palestinian | Israel |
| HGDP00677 | Palestinian | Israel |
| HGDP00689 | Palestinian | Israel |
| HGDP00678 | Palestinian | Israel |
| HGDP00690 | Palestinian | Israel |
| HGDP00679 | Palestinian | Israel |
| HGDP00691 | Palestinian | Israel |
| HGDP00680 | Palestinian | Israel |
| HGDP00692 | Palestinian | Israel |
| HGDP00693 | Palestinian | Israel |
| HGDP00683 | Palestinian | Israel |
| HGDP00682 | Palestinian | Israel |
| HGDP00694 | Palestinian | Israel |
| HGDP00725 | Palestinian | Israel |
| HGDP00736 | Palestinian | Israel |
| HGDP00684 | Palestinian | Israel |
| HGDP00696 | Palestinian | Israel |
| HGDP00727 | Palestinian | Israel |
| HGDP00738 | Palestinian | Israel |
| HGDP00685 | Palestinian | Israel |
| HGDP00739 | Palestinian | Israel |
| HGDP00686 | Palestinian | Israel |
| HGDP00699 | Palestinian | Israel |
| HGDP00731 | Palestinian | Israel |
| HGDP00700 | Palestinian | Israel |
| HGDP00732 | Palestinian | Israel |
| HGDP00744 | Palestinian | Israel |
| HGDP00722 | Palestinian | Israel |
| HGDP00733 | Palestinian | Israel |
| HGDP00745 | Palestinian | Israel |
| HGDP00723 | Palestinian | Israel |
| HGDP00734 | Palestinian | Israel |
| HGDP00746 | Palestinian | Israel |
| HGDP00724 | Palestinian | Israel |
| HGDP00735 | Palestinian | Israel |
| HGDP00740 | Palestinian | Israel |
| HGDP00726 | Palestinian | Israel |
| HGDP00737 | Palestinian | Israel |
| HGDP00234 | Pathan | Pakistan |
| HGDP00262 | Pathan | Pakistan |
| HGDP00213 | Pathan | Pakistan |
| HGDP00237 | Pathan | Pakistan |
| HGDP00264 | Pathan | Pakistan |
| HGDP00214 | Pathan | Pakistan |
| HGDP00239 | Pathan | Pakistan |
| HGDP00216 | Pathan | Pakistan |
| HGDP00241 | Pathan | Pakistan |
| HGDP00244 | Pathan | Pakistan |
| HGDP00228 | Pathan | Pakistan |
| HGDP00254 | Pathan | Pakistan |
| HGDP00230 | Pathan | Pakistan |
| HGDP00258 | Pathan | Pakistan |
| HGDP00232 | Pathan | Pakistan |
| HGDP00259 | Pathan | Pakistan |
| HGDP00218 | Pathan | Pakistan |
| HGDP00243 | Pathan | Pakistan |
| HGDP00222 | Pathan | Pakistan |
| HGDP00224 | Pathan | Pakistan |
| HGDP00226 | Pathan | Pakistan |
| HGDP00251 | Pathan | Pakistan |
| Romania1 | Romanian | Romania |
| Romania2 | Romanian | Romania |
| Romania3 | Romanian | Romania |
| Romania4 | Romanian | Romania |
| Romania5 | Romanian | Romania |
| Romania6 | Romanian | Romania |
| Romania7 | Romanian | Romania |
| Romania8 | Romanian | Romania |
| Romania9 | Romanian | Romania |
| Romania10 | Romanian | Romania |
| Romania11 | Romanian | Romania |
| Romania12 | Romanian | Romania |
| Romania13 | Romanian | Romania |
| Romania14 | Romanian | Romania |
| Romania15 | Romanian | Romania |
| Romania16 | Romanian | Romania |
| evo_1 | Russian | Estonia |
| evo_2 | Russian | Estonia |
| HGDP00880 | Russian | Russia |
| HGDP00892 | Russian | Russia |
| HGDP00879 | Russian | Russia |
| HGDP00891 | Russian | Russia |
| HGDP00903 | Russian | Russia |
| HGDP00890 | Russian | Russia |
| HGDP00902 | Russian | Russia |
| HGDP00881 | Russian | Russia |
| HGDP00893 | Russian | Russia |
| HGDP00882 | Russian | Russia |
| HGDP00894 | Russian | Russia |
| HGDP00884 | Russian | Russia |
| HGDP00896 | Russian | Russia |
| HGDP00885 | Russian | Russia |
| HGDP00897 | Russian | Russia |
| HGDP00886 | Russian | Russia |
| HGDP00898 | Russian | Russia |
| HGDP00887 | Russian | Russia |
| HGDP00899 | Russian | Russia |
| HGDP00883 | Russian | Russia |
| HGDP00895 | Russian | Russia |
| HGDP00888 | Russian | Russia |
| HGDP00900 | Russian | Russia |
| HGDP00901 | Russian | Russia |
| HGDP00889 | Russian | Russia |
| Samaritian983 | Samaritan | Israel |
| Samaritian988 | Samaritan | Israel |
| Samaritian990 | Samaritan | Israel |
| HGDP00991 | San | Namibia |
| HGDP01029 | San | Namibia |
| HGDP01032 | San | Namibia |
| HGDP00992 | San | Namibia |
| HGDP01036 | San | Namibia |
| HGDP00667 | Sardinian | Italy |
| HGDP01066 | Sardinian | Italy |
| HGDP01078 | Sardinian | Italy |
| HGDP00668 | Sardinian | Italy |
| HGDP01067 | Sardinian | Italy |
| HGDP01079 | Sardinian | Italy |
| HGDP00669 | Sardinian | Italy |
| HGDP01068 | Sardinian | Italy |
| HGDP01063 | Sardinian | Italy |
| HGDP01075 | Sardinian | Italy |
| HGDP00672 | Sardinian | Italy |
| HGDP01071 | Sardinian | Italy |
| HGDP00666 | Sardinian | Italy |
| HGDP01065 | Sardinian | Italy |
| HGDP01077 | Sardinian | Italy |
| HGDP00673 | Sardinian | Italy |
| HGDP01072 | Sardinian | Italy |
| HGDP00674 | Sardinian | Italy |
| HGDP01073 | Sardinian | Italy |
| HGDP01062 | Sardinian | Italy |
| HGDP01074 | Sardinian | Italy |
| HGDP00665 | Sardinian | Italy |
| HGDP01064 | Sardinian | Italy |
| HGDP01076 | Sardinian | Italy |
| HGDP00670 | Sardinian | Italy |
| HGDP01069 | Sardinian | Italy |
| HGDP01070 | Sardinian | Italy |
| HGDP00671 | Sardinian | Italy |
| SaudiA1 | Saudi | Saudi Arabia |
| SaudiA2 | Saudi | Saudi Arabia |
| SaudiA3 | Saudi | Saudi Arabia |
| SaudiA4 | Saudi | Saudi Arabia |
| SaudiA5 | Saudi | Saudi Arabia |
| SaudiA6 | Saudi | Saudi Arabia |
| SaudiA7 | Saudi | Saudi Arabia |
| SaudiA8 | Saudi | Saudi Arabia |
| SaudiA9 | Saudi | Saudi Arabia |
| SaudiA10 | Saudi | Saudi Arabia |
| saudi1411 | Saudi | Saudi Arabia |
| saudi1434 | Saudi | Saudi Arabia |
| saudi1403 | Saudi | Saudi Arabia |
| saudi1432 | Saudi | Saudi Arabia |
| saudi1424 | Saudi | Saudi Arabia |
| saudi1428b | Saudi | Saudi Arabia |
| saudi1430 | Saudi | Saudi Arabia |
| saudi1426 | Saudi | Saudi Arabia |
| saudi1436 | Saudi | Saudi Arabia |
| saudi1413 | Saudi | Saudi Arabia |
| sephardic12bul | Sephardic Jew | Bulgaria |
| sephardic13bul | Sephardic Jew | Bulgaria |
| sephardic14bul | Sephardic Jew | Bulgaria |
| sephardic15bul | Sephardic Jew | Bulgaria |
| sephardic16bul | Sephardic Jew | Bulgaria |
| sephardic17bul | Sephardic Jew | Bulgaria |
| sephardic18bul | Sephardic Jew | Bulgaria |
| sephardic19bul | Sephardic Jew | Bulgaria |
| sephardic11bel | Sephardic Jew | Spain |
| sephardic1tur | Sephardic Jew | Turkey |
| sephardic2tur | Sephardic Jew | Turkey |
| sephardic3tur | Sephardic Jew | Turkey |
| sephardic4tur | Sephardic Jew | Turkey |
| sephardic5tur | Sephardic Jew | Turkey |
| sephardic6tur | Sephardic Jew | Turkey |
| sephardic7tur | Sephardic Jew | Turkey |
| sephardic8tur | Sephardic Jew | Turkey |
| sephardic9tur | Sephardic Jew | Turkey |
| sephardic10tur | Sephardic Jew | Turkey |
| HGDP01335 | She | China |
| HGDP01336 | She | China |
| HGDP01327 | She | China |
| HGDP01334 | She | China |
| HGDP01328 | She | China |
| HGDP01329 | She | China |
| HGDP01332 | She | China |
| HGDP01331 | She | China |
| HGDP01333 | She | China |
| HGDP01330 | She | China |
| HGDP00163 | Sindhi | Pakistan |
| HGDP00187 | Sindhi | Pakistan |
| HGDP00210 | Sindhi | Pakistan |
| HGDP00165 | Sindhi | Pakistan |
| HGDP00189 | Sindhi | Pakistan |
| HGDP00167 | Sindhi | Pakistan |
| HGDP00191 | Sindhi | Pakistan |
| HGDP00169 | Sindhi | Pakistan |
| HGDP00192 | Sindhi | Pakistan |
| HGDP00173 | Sindhi | Pakistan |
| HGDP00197 | Sindhi | Pakistan |
| HGDP00181 | Sindhi | Pakistan |
| HGDP00205 | Sindhi | Pakistan |
| HGDP00183 | Sindhi | Pakistan |
| HGDP00206 | Sindhi | Pakistan |
| HGDP00185 | Sindhi | Pakistan |
| HGDP00208 | Sindhi | Pakistan |
| HGDP00171 | Sindhi | Pakistan |
| HGDP00195 | Sindhi | Pakistan |
| HGDP00175 | Sindhi | Pakistan |
| HGDP00199 | Sindhi | Pakistan |
| HGDP00177 | Sindhi | Pakistan |
| HGDP00201 | Sindhi | Pakistan |
| HGDP00179 | Sindhi | Pakistan |
| A382 | Southern Indian | India |
| D36 | Southern Indian | India |
| KNTK386 | Southern Indian | India |
| SAKD60 | Southern Indian | India |
| KNTK388 | Southern Indian | India |
| PNYD9 | Southern Indian | India |
| SAKD72 | Southern Indian | India |
| MLYA383 | Southern Indian | India |
| PNYD3 | Southern Indian | India |
| SAKD75 | Southern Indian | India |
| KNTK385 | Southern Indian | India |
| SAKD64 | Southern Indian | India |
| PNYD1 | Southern Indian | India |
| KNTK390 | Southern Indian | India |
| KNTK394 | Southern Indian | India |
| KNTK408 | Southern Indian | India |
| KNTK400 | Southern Indian | India |
| KNTK397 | Southern Indian | India |
| KNTK392 | Southern Indian | India |
| Spain8 | Spanish | Spain, Andalusia |
| Spain9 | Spanish | Spain, Andalusia |
| Spain10 | Spanish | Spain, Andalusia |
| Spain11 | Spanish | Spain, Andalusia |
| Spain12 | Spanish | Spain, Andalusia |
| Spain13 | Spanish | Spain, Andalusia |
| Spain1 | Spanish | Spain, Catalonia |
| Spain2 | Spanish | Spain, Catalonia |
| Spain3 | Spanish | Spain, Catalonia |
| Spain4 | Spanish | Spain, Catalonia |
| Spain5 | Spanish | Spain, Catalonia |
| Spain6 | Spanish | Spain, Catalonia |
| syria1 | Syrian | Syria |
| syria2 | Syrian | Syria |
| syria3 | Syrian | Syria |
| syria4 | Syrian | Syria |
| syria5 | Syrian | Syria |
| syria6 | Syrian | Syria |
| syria7 | Syrian | Syria |
| syria8 | Syrian | Syria |
| syria9 | Syrian | Syria |
| syria10 | Syrian | Syria |
| syria298 | Syrian | Syria |
| syria464 | Syrian | Syria |
| syria520 | Syrian | Syria |
| syria461 | Syrian | Syria |
| syria361 | Syrian | Syria |
| syria485 | Syrian | Syria |
| HGDP01347 | Tu | China |
| HGDP01348 | Tu | China |
| HGDP01349 | Tu | China |
| HGDP01350 | Tu | China |
| HGDP01351 | Tu | China |
| HGDP01352 | Tu | China |
| HGDP01353 | Tu | China |
| HGDP01356 | Tu | China |
| HGDP01355 | Tu | China |
| HGDP01354 | Tu | China |
| HGDP01101 | Tujia | China |
| HGDP01102 | Tujia | China |
| HGDP01100 | Tujia | China |
| HGDP01097 | Tujia | China |
| HGDP01098 | Tujia | China |
| HGDP01099 | Tujia | China |
| HGDP01104 | Tujia | China |
| HGDP01095 | Tujia | China |
| HGDP01096 | Tujia | China |
| HGDP01103 | Tujia | China |
| tur52 | Turk | Turkey |
| tur67 | Turk | Turkey |
| tur84 | Turk | Turkey |
| tur110 | Turk | Turkey |
| tur124 | Turk | Turkey |
| tur139 | Turk | Turkey |
| tur154 | Turk | Turkey |
| tur170 | Turk | Turkey |
| tur182 | Turk | Turkey |
| tur197 | Turk | Turkey |
| tur210 | Turk | Turkey |
| tur222 | Turk | Turkey |
| tur236 | Turk | Turkey |
| tur262 | Turk | Turkey |
| tur277 | Turk | Turkey |
| tur306 | Turk | Turkey |
| tur2 | Turk | Turkey |
| tur20 | Turk | Turkey |
| tur37 | Turk | Turkey |
| HGDP01167 | Italian | Italy, Tuscany |
| HGDP01162 | Italian | Italy, Tuscany |
| HGDP01163 | Italian | Italy, Tuscany |
| HGDP01169 | Italian | Italy, Tuscany |
| HGDP01164 | Italian | Italy, Tuscany |
| HGDP01166 | Italian | Italy, Tuscany |
| HGDP01168 | Italian | Italy, Tuscany |
| HGDP01305 | Uygur | China |
| HGDP01306 | Uygur | China |
| HGDP01304 | Uygur | China |
| HGDP01301 | Uygur | China |
| HGDP01302 | Uygur | China |
| HGDP01303 | Uygur | China |
| HGDP01298 | Uygur | China |
| HGDP01299 | Uygur | China |
| HGDP01297 | Uygur | China |
| HGDP01300 | Uygur | China |
| Uzbekistan_Jew_1 | Uzbek Jew | Uzbekistan |
| Uzbekistan_Jew_2 | Uzbek Jew | Uzbekistan |
| usb2 | Uzbek | Uzbekistan |
| usb6 | Uzbek | Uzbekistan |
| usb8 | Uzbek | Uzbekistan |
| usb13 | Uzbek | Uzbekistan |
| usb1 | Uzbek | Uzbekistan |
| usb25 | Uzbek | Uzbekistan |
| usb35 | Uzbek | Uzbekistan |
| usb78 | Uzbek | Uzbekistan |
| usb83 | Uzbek | Uzbekistan |
| usb16 | Uzbek | Uzbekistan |
| usb24 | Uzbek | Uzbekistan |
| usb32 | Uzbek | Uzbekistan |
| usb40 | Uzbek | Uzbekistan |
| usb64 | Uzbek | Uzbekistan |
| usb72 | Uzbek | Uzbekistan |
| HGDP01249 | Xibo | China |
| HGDP01250 | Xibo | China |
| HGDP01248 | Xibo | China |
| HGDP01245 | Xibo | China |
| HGDP01246 | Xibo | China |
| HGDP01247 | Xibo | China |
| HGDP01243 | Xibo | China |
| HGDP01251 | Xibo | China |
| HGDP01244 | Xibo | China |
| HGDP00946 | Yakut | Russia |
| HGDP00958 | Yakut | Russia |
| HGDP00947 | Yakut | Russia |
| HGDP00959 | Yakut | Russia |
| HGDP00948 | Yakut | Russia |
| HGDP00960 | Yakut | Russia |
| HGDP00949 | Yakut | Russia |
| HGDP00961 | Yakut | Russia |
| HGDP00950 | Yakut | Russia |
| HGDP00962 | Yakut | Russia |
| HGDP00945 | Yakut | Russia |
| HGDP00957 | Yakut | Russia |
| HGDP00969 | Yakut | Russia |
| HGDP00951 | Yakut | Russia |
| HGDP00963 | Yakut | Russia |
| HGDP00952 | Yakut | Russia |
| HGDP00964 | Yakut | Russia |
| HGDP00955 | Yakut | Russia |
| HGDP00967 | Yakut | Russia |
| HGDP00954 | Yakut | Russia |
| HGDP00966 | Yakut | Russia |
| HGDP00956 | Yakut | Russia |
| HGDP00968 | Yakut | Russia |
| HGDP00965 | Yakut | Russia |
| HGDP00953 | Yakut | Russia |
| Yemen_Jew_1 | Yemenite Jew | Yemen |
| Yemen_Jew_2 | Yemenite Jew | Yemen |
| Yemen_Jew_3 | Yemenite Jew | Yemen |
| Yemen_Jew_4 | Yemenite Jew | Yemen |
| Yemen_Jew_5 | Yemenite Jew | Yemen |
| Yemen_Jew_6 | Yemenite Jew | Yemen |
| Yemen_Jew_7 | Yemenite Jew | Yemen |
| Yemen_Jew_8 | Yemenite Jew | Yemen |
| Yemen_Jew_9 | Yemenite Jew | Yemen |
| Yemen_Jew_10 | Yemenite Jew | Yemen |
| Yemen_Jew_11 | Yemenite Jew | Yemen |
| Yemen_Jew_12 | Yemenite Jew | Yemen |
| Yemen_Jew_13 | Yemenite Jew | Yemen |
| Yemen_Jew_14 | Yemenite Jew | Yemen |
| Yemen_Jew_15 | Yemenite Jew | Yemen |
| Yemen1 | Yemenite | Yemen |
| Yemen2 | Yemenite | Yemen |
| Yemen3 | Yemenite | Yemen |
| Yemen4 | Yemenite | Yemen |
| Yemen5 | Yemenite | Yemen |
| Yemen6 | Yemenite | Yemen |
| Yemen7 | Yemenite | Yemen |
| Yemen8 | Yemenite | Yemen |
| Yemen9 | Yemenite | Yemen |
| Yemen10 | Yemenite | Yemen |
| HGDP01186 | Yizu | China |
| HGDP01187 | Yizu | China |
| HGDP01185 | Yizu | China |
| HGDP01182 | Yizu | China |
| HGDP01183 | Yizu | China |
| HGDP01184 | Yizu | China |
| HGDP01180 | Yizu | China |
| HGDP01181 | Yizu | China |
| HGDP01188 | Yizu | China |
| HGDP01179 | Yizu | China |
| HGDP00927 | Yoruba | Nigeria |
| HGDP00928 | Yoruba | Nigeria |
| HGDP00937 | Yoruba | Nigeria |
| HGDP00938 | Yoruba | Nigeria |
| HGDP00929 | Yoruba | Nigeria |
| HGDP00939 | Yoruba | Nigeria |
| HGDP00930 | Yoruba | Nigeria |
| HGDP00940 | Yoruba | Nigeria |
| HGDP00931 | Yoruba | Nigeria |
| HGDP00941 | Yoruba | Nigeria |
| HGDP00920 | Yoruba | Nigeria |
| HGDP00932 | Yoruba | Nigeria |
| HGDP00942 | Yoruba | Nigeria |
| HGDP00933 | Yoruba | Nigeria |
| HGDP00943 | Yoruba | Nigeria |
| HGDP00935 | Yoruba | Nigeria |
| HGDP00934 | Yoruba | Nigeria |
| HGDP00924 | Yoruba | Nigeria |
| HGDP00936 | Yoruba | Nigeria |
| HGDP00925 | Yoruba | Nigeria |
| HGDP00926 | Yoruba | Nigeria |

Table S2

A summary of the Jewish communities analyzed in this study

| Community | Samples | Sources |
| --- | --- | --- |
| Algerian | 5 | Behar et al. 2013 |
| Ethiopian | 23 | Behar et al. 2010, Lazaridis et al. 2014 |
| German | 2 | Behar et al. 2010 |
| Latvian | 2 | Behar et al. 2010 |
| Libyan | 15 | Behar et al. 2013, Lazaridis et al. 2014 |
| Moroccan | 26 | Behar et al. 2010, Behar et al. 2013, Lazaridis et al. 2014 |
| Tunisian | 13 | Behar et al. 2013, Lazaridis et al. 2014 |
| Austrian | 3 | Behar et al. 2010 |
| Belarusian | 2 | Behar et al. 2010 |
| Bulgarian | 8 | Behar et al. 2010 |
| Dutch | 3 | Behar et al. 2010 |
| French | 7 | Behar et al. 2010, Behar et al. 2013 |
| Italian | 10 | Behar et al. 2013 |
| Polish | 10 | Behar et al. 2010, Lazaridis et al. 2014 |
| Romanian | 3 | Behar et al. 2010 |
| Azerbaijani | 11 | Behar et al. 2010, Behar et al. 2013 |
| Georgian | 14 | Behar et al. 2010, Behar et al. 2013, Lazaridis et al. 2014 |
| Iranian | 21 | Behar et al. 2010, Behar et al. 2013, Lazaridis et al. 2014 |
| Iraqi | 20 | Behar et al. 2010, Behar et al. 2013, Lazaridis et al. 2014 |
| Kurdish | 10 | Behar et al. 2013 |
| Syrian | 2 | Behar et al. 2013 |
| Turkish | 21 | Behar et al. 2010, Behar et al. 2013, Lazaridis et al. 2014 |
| Uzbekistani | 5 | Behar et al. 2010, Behar et al. 2013 |
| Bnei Menashe | 6 | Behar et al. 2010, Behar et al. 2013 |
| Cochin | 12 | Behar et al. 2010, Behar et al. 2013 |
| Yemenite | 26 | Behar et al. 2010, Behar et al. 2013, Lazaridis et al. 2014 |

Table S3

Jewish individuals analyzed in this study

| **Pop Name** | **Code** | **Paper** |
| --- | --- | --- |
| Algerian Jew | AlgeriaJew1561 | Behar et al. 2013 |
| Algerian Jew | AlgeriaJew1855 | Behar et al. 2013 |
| Algerian Jew | AlgeriaJew4787 | Behar et al. 2013 |
| Algerian Jew | AlgeriaJew5183 | Behar et al. 2013 |
| Algerian Jew | AlgeriaJew5216 | Behar et al. 2013 |
| German Jew | ashkenazy6w | Behar et al. 2013 |
| German Jew | ashkenazy7w | Behar et al. 2013 |
| Latvian Jew | ashkenazy3e | Behar et al. 2013 |
| Latvian Jew | ashkenazy4e | Behar et al. 2013 |
| Dutch Jew | ashkenazy8w | Behar et al. 2013 |
| Dutch Jew | ashkenazy9w | Behar et al. 2013 |
| Dutch Jew | ashkenazy10w | Behar et al. 2013 |
| Polish Jew | AshkenaziJew5704 | Lazaridis et al. 2014 |
| Polish Jew | AshkenaziJew5728 | Lazaridis et al. 2014 |
| Polish Jew | AshkenaziJew5779 | Lazaridis et al. 2014 |
| Polish Jew | AshkenaziJew5782 | Lazaridis et al. 2014 |
| Polish Jew | AshkenaziJew5783 | Lazaridis et al. 2014 |
| Polish Jew | AshkenaziJew5788 | Lazaridis et al. 2014 |
| Polish Jew | AshkenaziJew5790 | Lazaridis et al. 2014 |
| Polish Jew | ashkenazy7e | Behar et al. 2010 |
| Polish Jew | ashkenazy8e | Behar et al. 2010 |
| Polish Jew | ashkenazy9e | Behar et al. 2010 |
| Romanian Jew | Romanian_Jew_1 | Behar et al. 2010 |
| Romanian Jew | Romanian_Jew_2 | Behar et al. 2010 |
| Romanian Jew | Romanian_Jew_3 | Behar et al. 2010 |
| Austria Jew | ashkenazy1w | Behar et al. 2010 |
| Austria Jew | ashkenazy2w | Behar et al. 2010 |
| Austria Jew | ashkenazy3w | Behar et al. 2010 |
| Belarusian Jew | ashkenazy1e | Behar et al. 2010 |
| Belarusian Jew | ashkenazy2e | Behar et al. 2010 |
| French Jew | FranceJewF38 | Behar et al. 2013 |
| French Jew | FranceJewF39 | Behar et al. 2013 |
| French Jew | FranceJewF48 | Behar et al. 2013 |
| French Jew | FranceJewF50 | Behar et al. 2013 |
| French Jew | FranceJewF58 | Behar et al. 2013 |
| French Jew | FranceJewF59 | Behar et al. 2013 |
| French Jew | ashkenazy4w | Behar et al. 2010 |
| Azerbaijani Jew | GeorgJewtat17 | Behar et al. 2010 |
| Azerbaijani Jew | GeorgJewtat16 | Behar et al. 2010 |
| Azerbaijani Jew | GeorgJewtat2 | Behar et al. 2010 |
| Azerbaijani Jew | GeorgJewtat14 | Behar et al. 2010 |
| Azerbaijani Jew | AzerbJew204 | Behar et al. 2010 |
| Azerbaijani Jew | AzerbJew210 | Behar et al. 2010 |
| Azerbaijani Jew | AzerbJew213 | Behar et al. 2010 |
| Azerbaijani Jew | AzerbJew218 | Behar et al. 2010 |
| Azerbaijani Jew | GRC12118114 | Behar et al. 2013 |
| Azerbaijani Jew | GRC12118116 | Behar et al. 2013 |
| Azerbaijani Jew | GRC12118123 | Behar et al. 2013 |
| Bnei Menashe Jew | Indian_Jew_5 | Behar et al. 2010 |
| Bnei Menashe Jew | Indian_Jew_6 | Behar et al. 2010 |
| Bnei Menashe Jew | Indian_Jew_7 | Behar et al. 2010 |
| Bnei Menashe Jew | Indian_Jew_8 | Behar et al. 2010 |
| Bnei Menashe Jew | GRC12118094 | Behar et al. 2013 |
| Bnei Menashe Jew | GRC12118106 | Behar et al. 2013 |
| Cochin Jew | Indian_Jew_1 | Behar et al. 2010 |
| Cochin Jew | Indian_Jew_2 | Behar et al. 2010 |
| Cochin Jew | Indian_Jew_3 | Behar et al. 2010 |
| Cochin Jew | Indian_Jew_4 | Behar et al. 2010 |
| Cochin Jew | GRC12118091 | Behar et al. 2013 |
| Cochin Jew | GRC12118096 | Behar et al. 2013 |
| Cochin Jew | GRC12118111 | Behar et al. 2013 |
| Cochin Jew | KuchinJew20320 | Lazaridis et al. 2014 |
| Cochin Jew | KuchinJew20551 | Lazaridis et al. 2014 |
| Cochin Jew | KuchinJew20622 | Lazaridis et al. 2014 |
| Cochin Jew | KuchinJew20851 | Lazaridis et al. 2014 |
| Cochin Jew | KuchinJew30052 | Lazaridis et al. 2014 |
| Ethiopian Jew | EthiopianJew1570 | Lazaridis et al. 2014 |
| Ethiopian Jew | EthiopianJew1574 | Lazaridis et al. 2014 |
| Ethiopian Jew | EthiopianJew1804 | Lazaridis et al. 2014 |
| Ethiopian Jew | EthiopianJew1818 | Lazaridis et al. 2014 |
| Ethiopian Jew | EthiopianJew1822 | Lazaridis et al. 2014 |
| Ethiopian Jew | EthiopianJew1831 | Lazaridis et al. 2014 |
| Ethiopian Jew | EthiopianJew4690 | Lazaridis et al. 2014 |
| Ethiopian Jew | eth_jew8 | Behar et al. 2010 |
| Ethiopian Jew | eth_jew10 | Behar et al. 2010 |
| Ethiopian Jew | eth_jew11 | Behar et al. 2010 |
| Ethiopian Jew | eth_jew12 | Behar et al. 2010 |
| Ethiopian Jew | eth_jew13 | Behar et al. 2010 |
| Ethiopian Jew | eth_jew14 | Behar et al. 2010 |
| Ethiopian Jew | eth_jew1 | Behar et al. 2010 |
| Ethiopian Jew | eth_jew2 | Behar et al. 2010 |
| Ethiopian Jew | eth_jew3 | Behar et al. 2010 |
| Ethiopian Jew | eth_jew4 | Behar et al. 2010 |
| Ethiopian Jew | eth_jew5 | Behar et al. 2010 |
| Ethiopian Jew | eth_jew6 | Behar et al. 2010 |
| Ethiopian Jew | eth_jew7 | Behar et al. 2010 |
| Ethiopian Jew | GRC12118092 | Behar et al. 2013 |
| Ethiopian Jew | GRC12118113 | Behar et al. 2013 |
| Ethiopian Jew | GRC12118119 | Behar et al. 2013 |
| Georgian Jew | GeorgJew105 | Behar et al. 2010 |
| Georgian Jew | GeorgJew111 | Behar et al. 2010 |
| Georgian Jew | GeorgJew125 | Behar et al. 2010 |
| Georgian Jew | GeorgJew141 | Behar et al. 2010 |
| Georgian Jew | GeorgianJew1577 | Lazaridis et al. 2014 |
| Georgian Jew | GeorgianJew1594 | Lazaridis et al. 2014 |
| Georgian Jew | GeorgianJew1607 | Lazaridis et al. 2014 |
| Georgian Jew | GeorgianJew1654 | Lazaridis et al. 2014 |
| Georgian Jew | GeorgianJew1671 | Lazaridis et al. 2014 |
| Georgian Jew | GeorgianJew1883 | Lazaridis et al. 2014 |
| Georgian Jew | GeorgianJew1971 | Lazaridis et al. 2014 |
| Georgian Jew | GRC12118107 | Behar et al. 2013 |
| Georgian Jew | GRC12118108 | Behar et al. 2013 |
| Georgian Jew | GRC12118125 | Behar et al. 2013 |
| Iranian Jew | IranianJew1132 | Lazaridis et al. 2014 |
| Iranian Jew | IranianJew1143 | Lazaridis et al. 2014 |
| Iranian Jew | IranianJew1159 | Lazaridis et al. 2014 |
| Iranian Jew | IranianJew1409 | Lazaridis et al. 2014 |
| Iranian Jew | IranianJew1513 | Lazaridis et al. 2014 |
| Iranian Jew | IranianJew1556 | Lazaridis et al. 2014 |
| Iranian Jew | IranianJew1557 | Lazaridis et al. 2014 |
| Iranian Jew | IranianJew1832 | Lazaridis et al. 2014 |
| Iranian Jew | IranianJew1845 | Lazaridis et al. 2014 |
| Iranian Jew | iran_jew1 | Behar et al. 2010 |
| Iranian Jew | iran_jew2 | Behar et al. 2010 |
| Iranian Jew | iran_jew3 | Behar et al. 2010 |
| Iranian Jew | iran_jew4 | Behar et al. 2010 |
| Iranian Jew | GRC12118101 | Behar et al. 2013 |
| Iranian Jew | GRC12118121 | Behar et al. 2013 |
| Iranian Jew | IranJew1409 | Behar et al. 2013 |
| Iranian Jew | IranJew1419 | Behar et al. 2013 |
| Iranian Jew | IranJew1425 | Behar et al. 2013 |
| Iranian Jew | IranJew1557 | Behar et al. 2013 |
| Iranian Jew | IranJew4485 | Behar et al. 2013 |
| Iranian Jew | IranJew4685 | Behar et al. 2013 |
| Iraqi Jew | iraq_jew1 | Behar et al. 2010 |
| Iraqi Jew | iraq_jew2 | Behar et al. 2010 |
| Iraqi Jew | iraq_jew3 | Behar et al. 2010 |
| Iraqi Jew | iraq_jew4 | Behar et al. 2010 |
| Iraqi Jew | iraq_jew5 | Behar et al. 2010 |
| Iraqi Jew | iraq_jew6 | Behar et al. 2010 |
| Iraqi Jew | iraq_jew7 | Behar et al. 2010 |
| Iraqi Jew | iraq_jew8 | Behar et al. 2010 |
| Iraqi Jew | iraq_jew9 | Behar et al. 2010 |
| Iraqi Jew | iraq_jew10 | Behar et al. 2010 |
| Iraqi Jew | iraq_jew11 | Behar et al. 2010 |
| Iraqi Jew | IraqiJew1417 | Lazaridis et al. 2014 |
| Iraqi Jew | IraqiJew1430 | Lazaridis et al. 2014 |
| Iraqi Jew | IraqiJew1771 | Lazaridis et al. 2014 |
| Iraqi Jew | IraqiJew4061 | Lazaridis et al. 2014 |
| Iraqi Jew | IraqiJew4241 | Lazaridis et al. 2014 |
| Iraqi Jew | IraqiJew4291 | Lazaridis et al. 2014 |
| Iraqi Jew | GRC12118082 | Behar et al. 2013 |
| Iraqi Jew | GRC12118102 | Behar et al. 2013 |
| Iraqi Jew | GRC12118129 | Behar et al. 2013 |
| Italian Jew | ItalyJew1 | Behar et al. 2013 |
| Italian Jew | ItalyJew10 | Behar et al. 2013 |
| Italian Jew | ItalyJew2 | Behar et al. 2013 |
| Italian Jew | ItalyJew3 | Behar et al. 2013 |
| Italian Jew | ItalyJew4 | Behar et al. 2013 |
| Italian Jew | ItalyJew5 | Behar et al. 2013 |
| Italian Jew | ItalyJew6 | Behar et al. 2013 |
| Italian Jew | ItalyJew7 | Behar et al. 2013 |
| Italian Jew | ItalyJew8 | Behar et al. 2013 |
| Italian Jew | ItalyJew9 | Behar et al. 2013 |
| Kurdish Jew | GRC12118100 | Behar et al. 2013 |
| Kurdish Jew | KurdJew1551 | Behar et al. 2013 |
| Kurdish Jew | KurdJew1580 | Behar et al. 2013 |
| Kurdish Jew | KurdJew1592 | Behar et al. 2013 |
| Kurdish Jew | KurdJew1824 | Behar et al. 2013 |
| Kurdish Jew | KurdJew4573 | Behar et al. 2013 |
| Kurdish Jew | KurdJew4633 | Behar et al. 2013 |
| Kurdish Jew | KurdJew4663 | Behar et al. 2013 |
| Kurdish Jew | KurdJew4689 | Behar et al. 2013 |
| Kurdish Jew | KurdJew856 | Behar et al. 2013 |
| Libyan Jew | LibyanJew1104 | Lazaridis et al. 2014 |
| Libyan Jew | LibyanJew1263 | Lazaridis et al. 2014 |
| Libyan Jew | LibyanJew1438 | Lazaridis et al. 2014 |
| Libyan Jew | LibyanJew1462 | Lazaridis et al. 2014 |
| Libyan Jew | LibyanJew1601 | Lazaridis et al. 2014 |
| Libyan Jew | LibyanJew1605 | Lazaridis et al. 2014 |
| Libyan Jew | LibyanJew1611 | Lazaridis et al. 2014 |
| Libyan Jew | LibyanJew1639 | Lazaridis et al. 2014 |
| Libyan Jew | LibyanJew1659 | Lazaridis et al. 2014 |
| Libyan Jew | LibyaJew1405 | Behar et al. 2013 |
| Libyan Jew | LibyaJew1439 | Behar et al. 2013 |
| Libyan Jew | LibyaJew1465 | Behar et al. 2013 |
| Libyan Jew | LibyaJew1579 | Behar et al. 2013 |
| Libyan Jew | LibyaJew1605 | Behar et al. 2013 |
| Libyan Jew | LibyaJew1611 | Behar et al. 2013 |
| Moroccan Jew | GRC12118098 | Behar et al. 2013 |
| Moroccan Jew | GRC12118103 | Behar et al. 2013 |
| Moroccan Jew | GRC12118104 | Behar et al. 2013 |
| Moroccan Jew | Morocco_Jew_1 | Behar et al. 2010 |
| Moroccan Jew | Morocco_Jew_2 | Behar et al. 2010 |
| Moroccan Jew | Morocco_Jew_3 | Behar et al. 2010 |
| Moroccan Jew | Morocco_Jew_4 | Behar et al. 2010 |
| Moroccan Jew | Morocco_Jew_5 | Behar et al. 2010 |
| Moroccan Jew | Morocco_Jew_6 | Behar et al. 2010 |
| Moroccan Jew | Morocco_Jew_7 | Behar et al. 2010 |
| Moroccan Jew | Morocco_Jew_8 | Behar et al. 2010 |
| Moroccan Jew | Morocco_Jew_9 | Behar et al. 2010 |
| Moroccan Jew | Morocco_Jew_10 | Behar et al. 2010 |
| Moroccan Jew | Morocco_Jew_11 | Behar et al. 2010 |
| Moroccan Jew | Morocco_Jew_12 | Behar et al. 2010 |
| Moroccan Jew | Morocco_Jew_13 | Behar et al. 2010 |
| Moroccan Jew | Morocco_Jew_14 | Behar et al. 2010 |
| Moroccan Jew | Morocco_Jew_15 | Behar et al. 2010 |
| Moroccan Jew | MorJew1973 | Behar et al. 2010 |
| Moroccan Jew | MorJew2001 | Behar et al. 2010 |
| Moroccan Jew | MoroccanJew4634 | Lazaridis et al. 2014 |
| Moroccan Jew | MoroccanJew4683 | Lazaridis et al. 2014 |
| Moroccan Jew | MoroccanJew4692 | Lazaridis et al. 2014 |
| Moroccan Jew | MoroccanJew4789 | Lazaridis et al. 2014 |
| Moroccan Jew | MoroccanJew5126 | Lazaridis et al. 2014 |
| Moroccan Jew | MoroccanJew5168 | Lazaridis et al. 2014 |
| Bulgaria Jew | sephardic12bul | Behar et al. 2010 |
| Bulgaria Jew | sephardic13bul | Behar et al. 2010 |
| Bulgaria Jew | sephardic14bul | Behar et al. 2010 |
| Bulgaria Jew | sephardic15bul | Behar et al. 2010 |
| Bulgaria Jew | sephardic16bul | Behar et al. 2010 |
| Bulgaria Jew | sephardic17bul | Behar et al. 2010 |
| Bulgaria Jew | sephardic18bul | Behar et al. 2010 |
| Bulgaria Jew | sephardic19bul | Behar et al. 2010 |
| Sephardic Jew | GRC12118097 | Behar et al. 2013 |
| Syrian Jew | SyriaJewK6 | Behar et al. 2013 |
| Syrian Jew | SyriaJewSY06 | Behar et al. 2013 |
| Tunisian Jew | TunisianJew1170 | Lazaridis et al. 2014 |
| Tunisian Jew | TunisianJew1421 | Lazaridis et al. 2014 |
| Tunisian Jew | TunisianJew1507 | Lazaridis et al. 2014 |
| Tunisian Jew | TunisianJew1511 | Lazaridis et al. 2014 |
| Tunisian Jew | TunisianJew1531 | Lazaridis et al. 2014 |
| Tunisian Jew | TunisianJew1544 | Lazaridis et al. 2014 |
| Tunisian Jew | TunisianJew1763 | Lazaridis et al. 2014 |
| Tunisian Jew | TunisiaJew1118 | Behar et al. 2013 |
| Tunisian Jew | TunisiaJew1421 | Behar et al. 2013 |
| Tunisian Jew | TunisiaJew1511 | Behar et al. 2013 |
| Tunisian Jew | TunisiaJew1544 | Behar et al. 2013 |
| Tunisian Jew | TunisiaJew5200 | Behar et al. 2013 |
| Tunisian Jew | TunisiaJew6074 | Behar et al. 2013 |
| Turkish Jew | GRC12118122 | Behar et al. 2013 |
| Turkish Jew | GRC12118128 | Behar et al. 2013 |
| Turkish Jew | sephardic1tur | Behar et al. 2010 |
| Turkish Jew | sephardic2tur | Behar et al. 2010 |
| Turkish Jew | sephardic3tur | Behar et al. 2010 |
| Turkish Jew | sephardic4tur | Behar et al. 2010 |
| Turkish Jew | sephardic5tur | Behar et al. 2010 |
| Turkish Jew | sephardic6tur | Behar et al. 2010 |
| Turkish Jew | sephardic7tur | Behar et al. 2010 |
| Turkish Jew | sephardic8tur | Behar et al. 2010 |
| Turkish Jew | sephardic9tur | Behar et al. 2010 |
| Turkish Jew | sephardic10tur | Behar et al. 2010 |
| Turkish Jew | SephardiJewTurkey4950 | Lazaridis et al. 2014 |
| Turkish Jew | SephardiJewTurkey4953 | Lazaridis et al. 2014 |
| Turkish Jew | SephardiJewTurkey4955 | Lazaridis et al. 2014 |
| Turkish Jew | SephardiJewTurkey5033 | Lazaridis et al. 2014 |
| Turkish Jew | SephardiJewTurkey5075 | Lazaridis et al. 2014 |
| Turkish Jew | SephardiJewTurkey5832 | Lazaridis et al. 2014 |
| Turkish Jew | SephardiJewTurkey5877 | Lazaridis et al. 2014 |
| Turkish Jew | SephardiJewTurkey6077 | Lazaridis et al. 2014 |
| Uzbekistani Jew | Uzbekistan_Jew_1 | Behar et al. 2010 |
| Uzbekistani Jew | Uzbekistan_Jew_2 | Behar et al. 2010 |
| Uzbekistani Jew | GRC12118089 | Behar et al. 2013 |
| Uzbekistani Jew | GRC12118109 | Behar et al. 2013 |
| Uzbekistani Jew | GRC12118115 | Behar et al. 2013 |
| Yemenite Jew | Yemen_Jew_1 | Behar et al. 2010 |
| Yemenite Jew | Yemen_Jew_2 | Behar et al. 2010 |
| Yemenite Jew | Yemen_Jew_3 | Behar et al. 2010 |
| Yemenite Jew | Yemen_Jew_4 | Behar et al. 2010 |
| Yemenite Jew | Yemen_Jew_5 | Behar et al. 2010 |
| Yemenite Jew | Yemen_Jew_6 | Behar et al. 2010 |
| Yemenite Jew | Yemen_Jew_7 | Behar et al. 2010 |
| Yemenite Jew | Yemen_Jew_8 | Behar et al. 2010 |
| Yemenite Jew | Yemen_Jew_9 | Behar et al. 2010 |
| Yemenite Jew | Yemen_Jew_10 | Behar et al. 2010 |
| Yemenite Jew | Yemen_Jew_11 | Behar et al. 2010 |
| Yemenite Jew | Yemen_Jew_12 | Behar et al. 2010 |
| Yemenite Jew | Yemen_Jew_13 | Behar et al. 2010 |
| Yemenite Jew | Yemen_Jew_14 | Behar et al. 2010 |
| Yemenite Jew | Yemen_Jew_15 | Behar et al. 2010 |
| Yemenite Jew | YemeniteJew4667 | Lazaridis et al. 2014 |
| Yemenite Jew | YemeniteJew4675 | Lazaridis et al. 2014 |
| Yemenite Jew | YemeniteJew4684 | Lazaridis et al. 2014 |
| Yemenite Jew | YemeniteJew4695 | Lazaridis et al. 2014 |
| Yemenite Jew | YemeniteJew4937 | Lazaridis et al. 2014 |
| Yemenite Jew | YemeniteJew4938 | Lazaridis et al. 2014 |
| Yemenite Jew | YemeniteJew4960 | Lazaridis et al. 2014 |
| Yemenite Jew | YemeniteJew5433 | Lazaridis et al. 2014 |
| Yemenite Jew | GRC12118086 | Behar et al. 2013 |
| Yemenite Jew | GRC12118087 | Behar et al. 2013 |
| Yemenite Jew | GRC12118095 | Behar et al. 2013 |
